# Longitudinal host-microbiome dynamics of metatranscription identify hallmarks of progression in periodontitis

**DOI:** 10.1186/s40168-025-02108-8

**Published:** 2025-05-14

**Authors:** Ana Duran-Pinedo, Jose O Solbiati, Flavia Teles, Zhang Yanping, Jorge Frias-Lopez

**Affiliations:** 1 Department of Oral Biology, University of Florida, College of Dentistry, 1395 Center Drive Gainesville, Gainesville, FL 32610 - 0424 USA; 2 Department of Basic & Translational Sciences, University of Pennsylvania, School of Dental Medicine, 240 South 40 Street, Philadelphia, PA 19104 - 6030 USA; 3 Center for Innovation and Precision Dentistry (CiPD), University of Pennsylvania, School of Dental Medicine, 240 South 40 Street, Philadelphia, PA 19104 - 6030 USA; 4 Gene Expression & Genotyping Core, Interdisciplinary Center for Biotechnology Research, University of Florida, 178 B CGRC, 2033 Mowry Road, Gainesville, FL 32610 USA

## Abstract

**Background:**

In periodontitis, the interplay between the host and microbiome generates a self-perpetuating cycle of inflammation of tooth-supporting tissues, potentially leading to tooth loss. Despite increasing knowledge of the phylogenetic compositional changes of the periodontal microbiome, the current understanding of in situ activities of the oral microbiome and the interactions among community members and with the host is still limited. Prior studies on the subgingival plaque metatranscriptome have been cross-sectional, allowing for only a snapshot of a highly variable microbiome, and do not include the transcriptome profiles from the host, a critical element in the progression of the disease.

**Results:**

To identify the host-microbiome interactions in the subgingival milieu that lead to periodontitis progression, we conducted a longitudinal analysis of the host-microbiome metatranscriptome from clinically stable and progressing sites in 15 participants over 1 year. Our research uncovered a distinct timeline of activities of microbial and host responses linked to disease progression, revealing a significant clinical and metabolic change point (the moment in time when the statistical properties of a time series change) at the 6-month mark of the study, with 1722 genes differentially expressed (DE) in the host and 111,705 in the subgingival microbiome. Genes associated with immune response, especially antigen presentation genes, were highly up-regulated in stable sites before the 6-month change point but not in the progressing sites. Activation of cobalamin, porphyrin, and motility in the microbiome contribute to the progression of the disease. Conversely, inhibition of lipopolysaccharide and glycosphingolipid biosynthesis in stable sites coincided with increased immune response. Correlation delay analysis revealed that the positive feedback loop of activities leading to progression consists of immune regulation and response activation in the host that leads to an increase in potassium ion transport and cobalamin biosynthesis in the microbiome, which in turn induces the immune response. Causality analysis identified two clusters of microbiome genes whose progression can accurately predict the outcomes at specific sites with high confidence (AUC = 0.98095 and 0.97619).

**Conclusions:**

A specific timeline of host-microbiome activities characterizes the progression of the disease. The metabolic activities of the dysbiotic microbiome and the host are responsible for the positive feedback loop of reciprocally reinforced interactions leading to progression and tissue destruction.

Video Abstract

**Supplementary Information:**

The online version contains supplementary material available at 10.1186/s40168-025-02108-8.

## Background

The oral microbiome ecosystem is critical to oral and systemic human health [1–3]. In periodontitis, a chronic disease affecting 47% of US adults, specific bacterial ecology elicits inflammatory responses in the host, leading to the destruction of periodontal tissue, pocket formation, and tooth loss [4, 5]. Interestingly, the connections between oral microbes and health extend beyond the oral cavity; many studies have shown that periodontal diseases influence the risk for certain systemic conditions [6–9]. Understanding the forces that lead to a dysbiotic oral microbiome causing periodontitis is crucial for understanding both oral and broader systemic health and improving the treatment of periodontitis.

Tissue destruction progresses through periods of acute exacerbation (activity) followed by periods of remission [10–13]. Initially formulated as the “ecological plaque hypothesis” by Marsh [14, 15], to explain periodontitis progression proposes that changes in the relative abundance of members of the oral microbiome lead to dysbiosis; thus, changes in the composition of subgingival biofilms potentially explaining periods of disease activity. Several studies have identified differences in the levels of subgingival species when comparing diseased and healthy sites [16–18]. However, these studies also revealed considerable overlap in the composition of the microbial communities regardless of health status, suggesting that changes in microbial composition may not entirely explain the differences in periodontal status. While specific pathogen species such as *Porphyromonas gingivalis* may dominate in periodontitis, other commensal species persist, suggesting that not all shifts in community composition lead to disease progression [19, 20]. This finding aligns with earlier works by Griffen et al., which stressed the intricate profiles of bacterial communities in periodontitis versus health, highlighting the functional capabilities of these taxa as critical distinguishing elements beyond their taxonomic identities [21].

While considerable advances have been made in understanding the complex ecological interactions, functional gene expression, and metabolic networks in the periodontal microbiome [19, 22–24], there remains a need to understand better how metabolic activity, rather than just community structure, correlates with disease progression. Recent studies suggest that signature metabolic activities may define periodontitis pathogenesis regardless of specific bacterial community composition [25–29], highlighting the importance of functional analysis alongside taxonomic profiling. The exacerbation of inflammation, leading to bone loss, is primarily driven by the host-microbiome crosstalk rather than the actions of specific organisms [4, 30]. While prior studies on the oral metatranscriptome have provided valuable insights, most have been cross-sectional, offering only a snapshot of a highly variable microbiome at a given time and failing to include the host transcriptome profiles, which are crucial to understanding disease progression [25, 27, 28, 31]. This gap in knowledge highlights the need for further research to understand better how a dysbiotic microbiome interacts with the host to disrupt its physiology, leading to inflammation and pathological bone resorption. The origins of dysbiosis, whether it is a cause or consequence of disease, remain unclear, and the precise roles of innate and adaptive immune components in the inflammatory dialogue between the host and microbiome in periodontitis are yet to be fully understood [32, 33].

The present study was part of a large longitudinal clinical trial that followed patients with periodontitis for 1 year [34, 35]. Our study aimed to elucidate the role of the dysbiotic oral microbiome and the host in the progression of periodontitis by analyzing two clinically distinct sets of teeth: one group comprising teeth that progressed in disease (showing significant loss of attachment to the gingiva) and the other group consisting of teeth that remained clinically stable, from the same patients. Using host-microbiome transcriptome analysis, we explored the contributions of both the dysbiotic microbiome and the host to disease progression. Our results reveal key metabolic activities that explain the dysbiotic cycle perpetuating inflammation and tissue destruction during the progression of periodontitis.

## Methods

A detailed description of all bioinformatic pipelines used in this paper is presented in the jupyter notebook “*Duran-Pinedo_et_al_notebook.ipynb*” as supporting information. When possible, GNU parallel was used in all bioinformatics analyses [36].

### Study design and population

Figure S1 details the study design and overall approach. The patient population consisted of 15 individuals presenting periodontitis Stage II/III. They represent a subset of a larger cohort devised to study periodontitis progression based on changes in clinical attachment. Two sites were chosen for each, each representing one of the clinical groups: stable and progressing (2 sites/participant, 30 sites total) (Fig. S1). All teeth selected for analysis in the present study were clinically identical at baseline.

The full study has been described previously [34, 35]. That cohort received comprehensive periodontal examination by calibrated examiners every 2 months for 12 months of delayed treatment to monitor for periodontal disease progression. The study followed a strict safety protocol, and further details can be obtained at ClinicalTrials.gov (https://clinicaltrials.gov/ct2/home) under the identifier NCT01489839. Because the original study aimed to identify biomarkers of periodontitis progression, periodontal treatment needed to be delayed so that data and samples of untreated and progressing sites could be collected and studied. While the study called for treatment delay, participants were closely monitored and protected. They received periodontal evaluation every 2 months, a frequency of evaluation greater than that typically seen in clinical dental practice. In addition, as part of the protocol, safety thresholds were carefully designed to monitor disease progression. Subjects with ≥ 6 sites with a cumulative loss of attachment ≥ 2 mm from baseline during the monitoring phase had their monitoring interrupted and proceeded to treatment. Participants displaying ≥ 4 mm of CAL increase at any individual site received periodontal rescue therapy only at impacted sites and continued with monitoring. The Forsyth Institutional Review Board reviewed and approved the study’s protocol, and each recruitment center approved it. In addition, the study was monitored by an NIH-provided CRO (Clinical Research Organization).

Details regarding patient recruitment, monitoring, and patterns of periodontitis progression have been published elsewhere [34, 35]. In brief, the clinical phase of the study (i.e., recruitment, enrollment, monitoring, and treatment) occurred between 2012 and 2016. Participants were recruited according to the following criteria: (a) mild periodontitis: ≥ 4 separate teeth with ≥ 1 site of PD ≥ 5 mm and concomitant CAL ≥ 2 mm, radiographic evidence of alveolar bone loss around ≥ 2 of the affected teeth; (b) severe periodontitis: ≥ 8 separate teeth with ≥ 1 site of PD ≥ 5 mm and concomitant CAL ≥ 3 mm; radiographic evidence of alveolar bone loss around ≥ 2 of the affected teeth. In addition, patients were ≥ 25 years old, with ≥ 20 natural teeth, of which ≥ 12 of these teeth were pre-molars, first or second molars. Exclusion criteria were periodontal or systemic antibiotic therapy in the previous 6 months, the use of tobacco products within 1 year before the screening visit, any medical condition that might influence the course of periodontal disease or treatment, and chronic use of NSAIDs. Participants were recruited at The Forsyth Institute (Cambridge, MA, USA), New York University College of Dentistry (New York, NY, USA), State University of New York at Buffalo School of Dental Medicine (Buffalo, NY, USA), and Southern Illinois University School of Dental Medicine (Alton, IL, USA).

Once all clinical data were obtained, compiled, and curated, statistical methods were used to model progression [35]. Then, this study’s sites and samples were selected for analysis based on that progression model that classified them as stable or progressing. A summary of the patient population and clinical measurements is presented in Table S1.

Subject recruitment and study procedures were approved by and carried out following the Institutional Review Board at The Forsyth Institute.

### Periodontal sites and subgingival plaque samples

After removing supragingival plaque, subgingival plaque samples were individually collected from each site with a single stroke using a sterile Gracey mini-curette. Each sample was placed in a separate microcentrifuge tube.

**Fig. 1:**
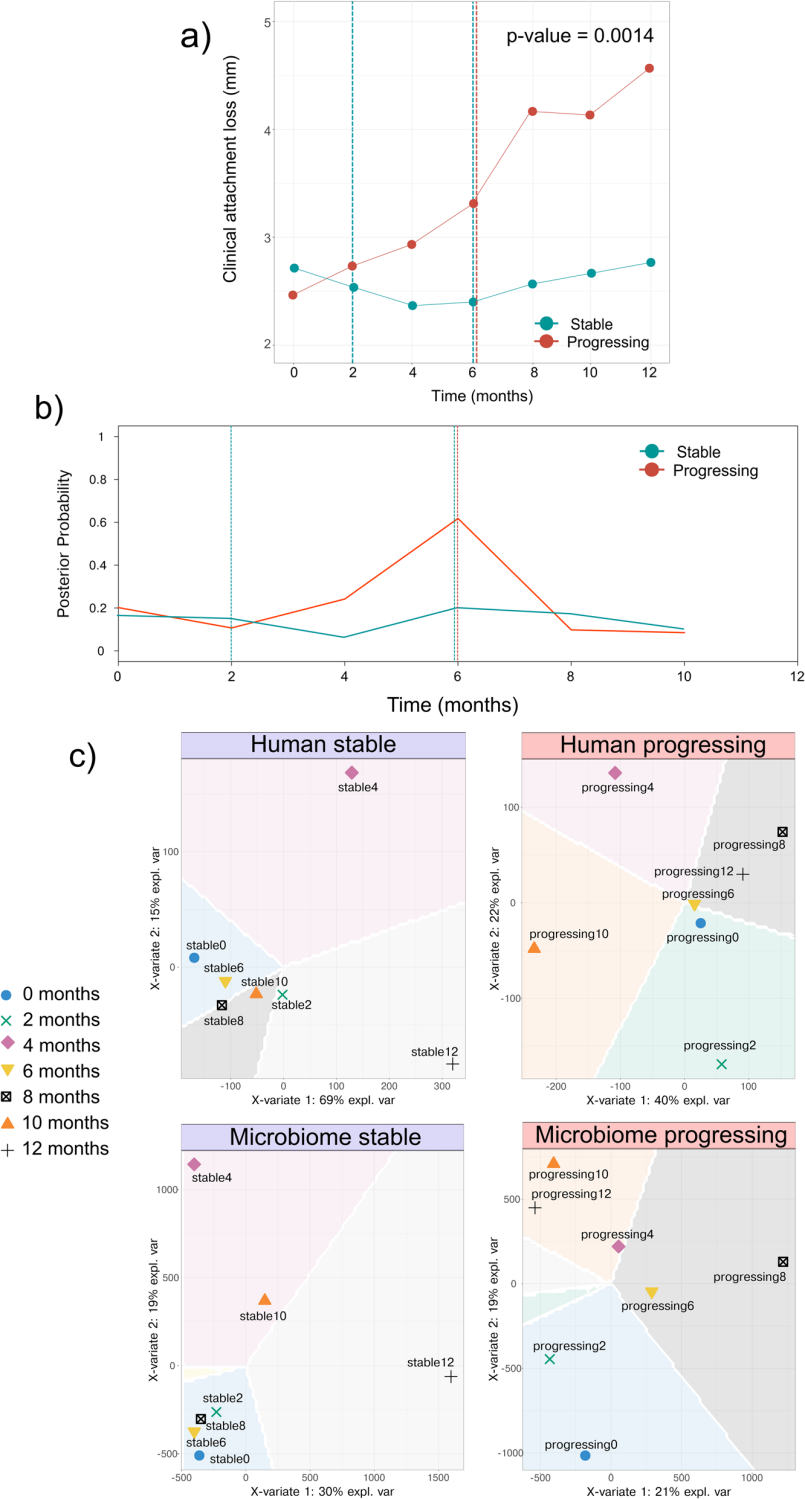
Detecting change points in the time series. a Observed results of CAL and change points in the stable and progressing time series based on mean and variance. b Posterior probabilities of a change point at each position. c sPLS-DA (sparse partial least squares discriminant analysis) classification of the whole transcriptome in the host and microbiome. The plots display the first two components from sparse partial least squares discriminant analysis, with explained variance percentages for Component 1 shown for each analysis (human stable: 69%, human progressing: 40%, microbiome stable: 30%, microbiome progressing: 21%). Samples are colored by group and shaped by timepoint (0–12 months), depicting the prediction background as a color-coded probability for each plot region. We used the default method (max.dist) for classification, which assigns classes based on the closest sample from the training set

From each of the 15 study participants, 2 sites were selected for Dual-RNA Seq analysis: 1 progressing site and 1 stable site. The two groups were devised based on linear mixed models (LMM) fitted to longitudinal CAL measurements for each site during the 12 months of monitoring. The predicted CAL levels were used to categorize sites regarding progression or regression. Two groups were defined based on the LMM results applied to CAL [35]: sites that showed disease progression and sites that remained clinically stable during the study. The actual profiles of CAL during the study and after treatment are shown in Fig. 1. All these sites were mesial or distal buccal sites of posterior teeth. After removing supragingival plaque, subgingival plaque samples were individually collected from each site with a single stroke using a sterile Gracey mini-curette. Each sample was placed in a separate microcentrifuge tube and immediately frozen. Samples were stored at − 80 °C until analysis.

### Change point analysis

First we assessed the significance of the differences between the tow CAL trajectories performing a permutation test for global trajectory differences using the R package “*coin*” [37]. To detect changes or divergence points in time-series data, we used the R package “*changepoint*” [38], which detects changes in mean, variance, or other properties of the time series that might correspond to divergence points. It employs penalized cost functions to determine the number and location of change points. To measure the confidence of the different breakpoints, we used the R package “*bcp*” (Bayesian change point) [39], which outputs posterior probabilities for each point being a change point. The output represents the 2.5% and 97.5% quantiles of the change points detected across all bootstrap samples, effectively providing a 95% confidence interval for the location of change points in the time series data. Finally, for a more controlled Bayesian analysis where we specify our own model, we use the R package “*rstan*” [40]. The length and direction of the arrow will tell you how significant the change is at the point and in which direction (increase or decrease).

### RNA extraction and sequencing

Total RNA was extracted as described by Duran-Pinedo et al. (A. E. Duran-Pinedo et al., 2014). Briefly, cells from the subgingival plaque samples were collected by centrifugation for 10 min at maximum speed in a microcentrifuge. Six hundred microliters of mirVana kit (Life Technologies) lysis/binding buffer and 300 μl of 0.1-mm zirconia-silica beads (BioSpec Products, Bartlesville, OK, USA) were added to the samples. The beads were cleaned and sterilized beforehand with HCl acid and bleach washes. Finally, the beads were treated with diethylpyrocarbonate overnight and autoclaved. Samples were bead beaten for 1 min at maximum speed. RNA was extracted following the protocol of mirVana™ miRNA Isolation Kit, with phenol Catalog number AM1560 (Thermofisher Scientific). Linear RNA amplification was performed on total bacterial RNA using MessageAmp™ II-Bacteria RNA Amplification Kit Catalog number AM1790 (Thermofisher Scientific). All kits were used following the manufacturer’s instructions. Then, the RNA-seq library is processed using NEBNext® Ultra™ Directional RNA Library Prep Kit for Illumina (NEB, USA), following the manufacturer’s recommendations. Five microliters of depleted RNA mix and 5 μl of the first-strand synthesis reaction mix, fragmented by heating at 94 °C for the desired time. This step is followed by first-strand cDNA synthesis using reverse transcriptase and oligo dT primers. Next, synthesis of ds cDNA is performed using the second strand master mix provided in the kit, followed by end-repair and adaptor ligation. Finally, the library is enriched by several amplification cycles and purified by Agencourt AMPure beads (Beckman Coulter, catalog #A63881).

Individual libraries were pooled with equimolar preparation for sequencing; barcoded libraries were sized on the Agilent 2200 TapeStation. Quantitation was done by QUBIT and qPCR (Kapa Biosystems, catalog number KK4824). Individual samples were pooled and input in the NovaSeq 6000 instrument. Typically, a 250-pM library concentration was used to cluster the cBOT, resulting in an optimum clustering density at which the percentage of clusters passing filters was 65–75%. Six RNA-Seq barcoded libraries were pooled for sequencing in multiplex on a single flow cell lane, using a 2 × 150 cycles (paired-end) configuration. A typical sequencing run in the NovaSeq 6000 S4 flow cell produced > 800 million reads from each end per lane with a Q30 > = 90%. RNA-seq library construction performed at UF ICBR Gene Expression & Genotyping (https://biotech.ufl.edu/gene-expression-genotyping/, RRID: SCR_019145). Sequencing was performed at ICBR NextGen (https://biotech.ufl.edu/next-gen-dna/, RRIC: SCR_019152).

### Host-microbiome metatranscriptome analysis: taxonomic profiling, differential expression, clustering trajectories, and gene set enrichment analysis

Sequences were cleaned up to remove low-quality sequences using *Trimmomatic* [41]. Cleaned sequences from both host and microbiome were aligned against their correspondent database indexes using STAR [42]. We generated a custom dabatase for the oral microbiome based on the species identified as part of the subgingival plaque ecosystem from the HOMD database [43]. The database consists of 990 genomes (strains) from 283 species. Based on the values for species abundance in the HOMD the species included in our database cover 99.053% of the total abundance [43]. The list of genomes and accession numbers are in Table S2.

For the analysis of the host transcriptome, sequence alignment was performed against Release 36 (GRCh38.p13) of the human genome (https://www.gencodegenes.org/human/release_36.html). Counts were obtained using *featureCounts* [44]. Details for all running conditions are presented in the jupyter notebook “*Duran-Pinedo_et_al_notebook.ipynb*”. The microbial genomes database used for taxonomic profiling was the same as the one used for metatranscriptome analysis. Phylogenetic assignment and relative quantification were performed using Kraken2 [45] and Bracken [46].

Differential Expression analysis was performed using the function *nbinomLRT* from the R package *DESeq2* [47] as recommended for short (< 8-time points) time-series analysis by Spies et al. [48].

For clustering trajectories, counts were normalized using the *zscore* function from the R package *dtwclust* [49]. The number of optimal clusters for clustering gene expression trajectories of differentially expressed (DE) genes was obtained using the *fviz_nbclust* function from the R package *factoextra* [50]. Clusters were obtained using the *tsclust* function from the R package *TSclust* [51].

To perform gene set enrichment analysis of the genes in each of the clusters, we used the Cytoscape app *ClueGO* [52]. The detailed parameters used in the analysis are described in the jupyter notebook “*Duran-Pinedo_et_al_notebook.ipynb*”. We generated a new organism database called “oral microbiome” inside the ClueGOConfiguration folder to gain access to the GO and KEGG ontologies for the oral microbiome and perform enrichment analysis. Given their high number, for the analysis of all DE genes in the host and the microbiome, we used the R packages *clusterProfiler *[53].

### Statistical analysis

To assess whether a sample size of 15 subjects per site group likely affords adequate statistical power, we calculated the effect size measured as omega-squared (ω^2^) described in Kelly et al. [54] using Jaccard distance. This method has been specifically designed to estimate sample size for microbiome analysis. We found that with a power of 90%, the stable group has a ω^2^ of 0.019 and the progressing group 0.042, all smaller than the ω^2^ of 0.08 that Kelly et al. found in the ten subjects per group. Power analysis was performed using the R package “*micropower*” [54].

Taxonomic representation of statistically consistent differences between the groups was performed using the linear discriminant analysis effect size (LEfSe) method, which allows for identifying biological biomarkers associated with a particular condition [55]. We used the default settings for the statistical tests: 0.05 as the α value for the factorial Kruskal–Wallis test among classes and for the pairwise Wilcoxon test between subclasses and a threshold of 2 on the logarithmic LDA score for discriminative features.

sPLS-DA is a statistical method for classification and feature selection in high-dimensional data, such as omics datasets. It is implemented in the mixOmics package in R [56], designed to analyze large biological datasets, including genomics, proteomics, metabolomics, and transcriptomics. sPLS-DA is a supervised method that uses class labels (e.g., disease vs. control) to guide the classification. The goal is to find the components (latent variables) that best separate the classes while also being sparse, meaning that only a subset of the original variables (e.g., genes, proteins, metabolites) are selected and used. Feature selection: one of the primary advantages of sPLS-DA is its ability to perform feature selection while constructing the model. It identifies the most relevant features contributing to class discrimination, which is particularly useful in high-dimensional datasets where the number of variables exceeds the number of samples.

## Results

### A clinical change point coincides with a change in the host-microbiome transcriptome

Figure 1a show the progression of CAL in both clinical groups in the patients analyzed. The tow trajectories are significantly different (*p*-value = 0.0014). Employing penalized cost functions to determine the number and location of change points [38], we found that the stable sites had one change at 2 months and another at 6 months, while the progressing sites had only one change point at 6 months (Fig. 1a). However, the analysis of posterior probabilities identified only the progressing 6-month change point as significant (Fig. 1b).

The defined change point in the clinical data coincides with a significant shift in the overall expression profiles of both the host and the microbiome. We used sparse partial least squares discriminant analysis (sPLS-DA) [56], a supervised classification method, to assess how the different transcriptomes changed with time. As shown in Fig. 1c, in the stable transcription profiles at month 4, there is a big shift in the profiles of the total transcriptome. After that, the transcriptomes return to a profile similar to the baseline. The profile at the last time point is also very different. However, in the progressing sites, after the shift from the baseline, the transcriptomes never go back to the initial baseline, probably associated with the irreversibility observed in the clinical data.

### A defined succession of bacterial complexes is associated with the clinical outcome of the periodontal site

**Fig. 2:**
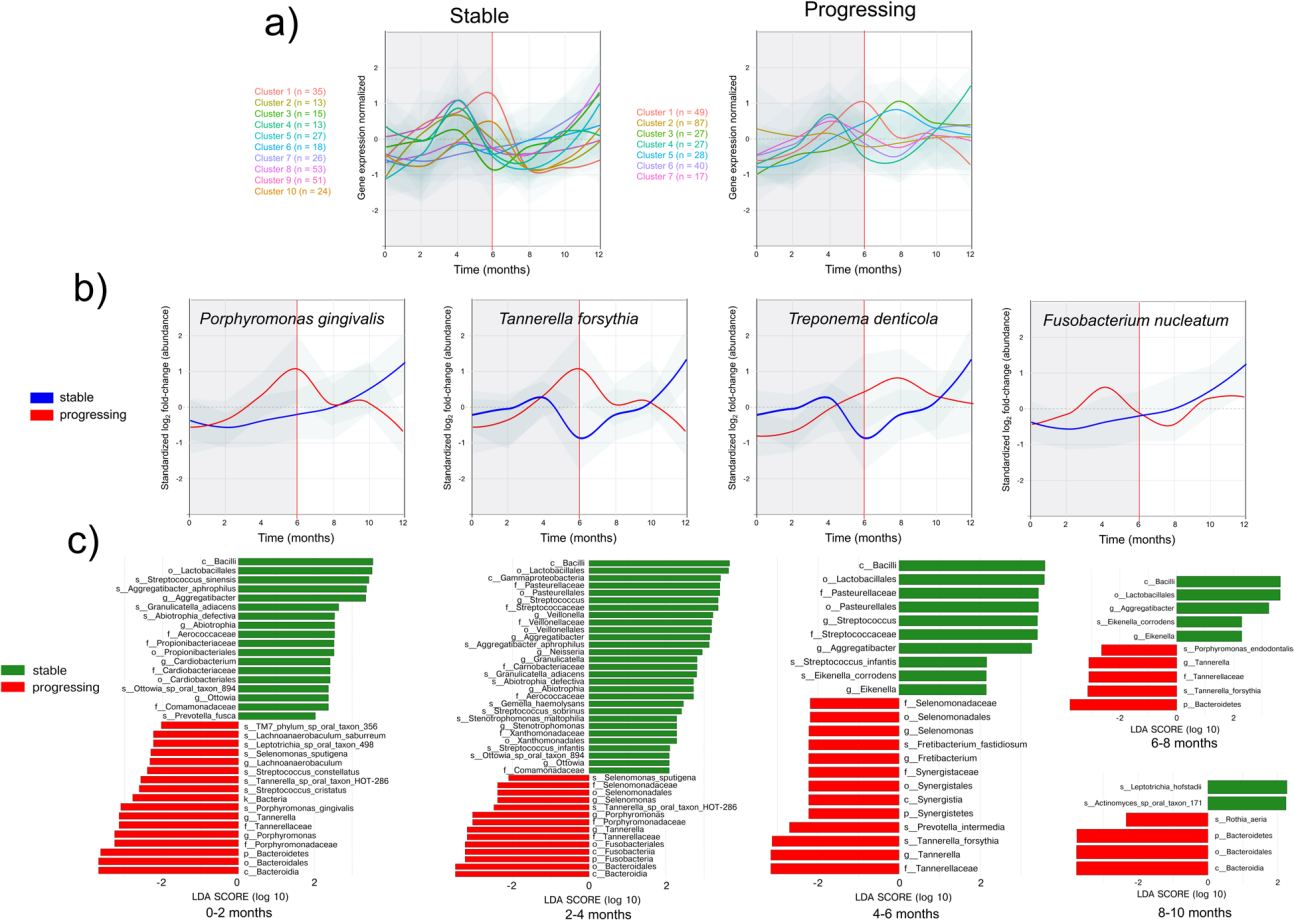
Phylogenetic assignment and relative quantification of microbiome metatranscriptome. a Clusters of microbial species obtained by determining an optimal number of clusters using fviz_nbclust from the ‘factoextra’ package with the gap statistic method and performing clustering using tsclust with shape-based distance (SBD), which makes the clustering particularly useful for time series where the shape matters more than exact numerical values. Clusters are standardized to log2 fold-change of abundance. Colors and cluster numbers are arbitrary. The actual composition of the different clusters is presented in Table S1. b Profiles of abundance of the periodontopathogens Porphyromonas gingivalis, Tannerella forsythia, Treponema denticola, and Fusobacterium nucleatum. c Temporal LEfSe analysis showing the distribution of taxonomic lineages within different samples at different time points as determined by linear discriminant analysis (LDA) effect size (LEfSe). Only taxons with LDA scores higher than two are shown

We first assigned transcripts to microbial species and identify the phylogenetic origin of the transcripts as a proxy for the composition of the active community. Once the assignment was performed, we clustered the trajectories of the different species to identify groups of species that followed the same dynamics during the study period. These clusters, which represent distinct patterns of microbial community dynamics, are crucial in understanding the changes in the oral microbiome over time (Fig. 2a). Ten clusters were identified in the stable samples (Table S3). Seven in the progressing sites, with different numbers of species in each cluster (Fig. 2a, Table S3). We observed that some of the clusters have a maximum peak before 6 months (Fig. S2a) and decided to combine them to determine the difference in composition in stable and progressing samples (Fig. S2a).

We observed species found in both conditions, but others were specific to the stable or progressing samples (Fig. S2b). A core group of 38 species that peaked before the sixth month was present in both stable and progressing samples, with *Leptotrichia* and *Prevotella* the most frequent genera, genera found previously in both healthy and diseased patients [57, 58] (Fig. S2c). Sixty-six species were only present in stable samples, with many members of the genera *Capnocytophaga* and *Actinomyces,* usually associated with health [57, 58] (Fig. S2c). Finally, species of the *Neisseria* and *Actinomyces* genera were abundant in the 46 species, with a maximum of 6 months only found in progressing sites.

Interestingly, *Fusobacterium nucleatum,* which is frequently one of the first pathogens to proliferate in the subgingival plaque during the transition disease [59]*,* was among the species that peaked during the first 6 months only in progressing sites, while in stable sites, it was present at low levels during the same period (Fig. 2b, S2c). Two major peridontopathogens, *P. gingivalis* and *Tannerella forsythia*, a group of organisms highly associated with severe disease [60, 61], peaked exactly at month 6 in progressing sites but were not abundant during that period (Fig. 2c). *Treponema denticola*, another important periodontal pathogen [60, 61] peaked later but steadily increased in proportion in progressing sites while being present in low numbers before the change point (Fig. 2c). We then used Linear discriminant analysis Effect Size (LEfSe) [55] to identify features taxa that are statistically different between conditions at different periods. In general, there was a tendency for reduction in the number of biomarkers in both the stable and progressing sites after the 4–6 months period (Fig. 2c). This reduction was even more marked after periods 6–8 and 8–10 months with no significant differences at the period 10 to 12 months (Fig. 2c). There were familiar elements between different periods. In the stable sites class Bacilli, order Lactobacillales, genus *Streptococcus*, and *Aggregatibacter* were all present in the periods from 0 to 6 months, while in the period 6 to 8 months, the genus *Streptococcus* was absent. Interestingly, *Granulicatella adjacent*, *Abiotrophia defectiva*, and genus *Ottowia* were biomarkers of the stable sites from 0 to 4 months, while disappearing after that and being substituted by *Eikenella corrodens*, an organism frequently associated with disease [62]*,* from 4 to 8 months (Fig. 2c). *Granulicatella adiacens* and *Abiotrophia defectiva* are integral components of the oral microbiome, with their roles oscillating between commensalism and pathogenicity depending on the host’s health status [63–66]. A pattern of similarities between 0 and 4 months was found in the progressing sites, with commonly associated periodontal pathogens [60, 61, 67] *Selenomonas*, *Porphyromonas*, and *Tannerella* genera being common markers for those periods. *Tannerella* genus remains a marker up to the 8 months while *Selenomonas* goes only up to the 4–6-month period.

### Longitudinal analysis of host-microbiome transcriptomes reveals hallmarks of periodontitis progression

The longitudinal data enabled the identification of temporal hallmarks that define the divergence between stable and progressing sites. We first analyzed the differences in gene expression on our time series and looked at global changes during the progression of the disease. Our analysis showed that 1722 genes were differentially expressed (DE) in the host and 111,705 in the oral microbiome (Table S4).

We used the DE genes in the host and microbiome to perform the KEGG pathway and gene ontology enrichment analysis on those genes. Host activities showed an overall activation of the inflammatory and adaptive immune responses (Fig. S3). Coincidentally, we also observed inhibition of ion transport, specifically potassium ion transport. In the microbiome, an inhibition of porphyrin synthesis involved in heme acquisition [68], suppression of branched-chain amino acids (BCAAs) valine, leucine, and isoleucine synthesis, and activation of glycine, serine, and threonine metabolism, probably indicating an adaptation to oxidative stress conditions [69, 70] (Fig. S3). Additionally, vitamin B_6_ and taurine and hypotaurine metabolism were also activated during the evolution of the disease.

**Fig. 3:**
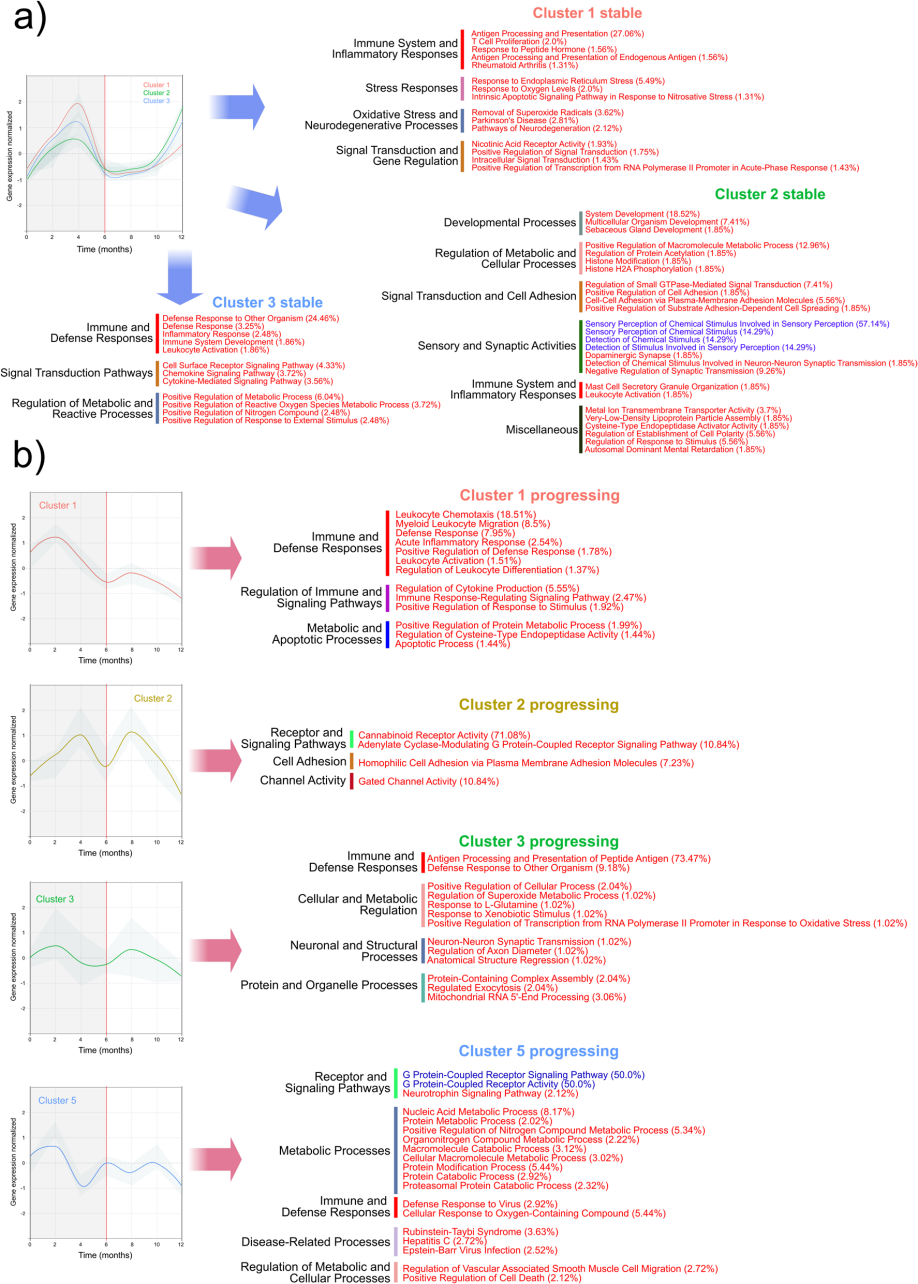
Human GO ontology and KEGG pathways enrichment analysis of differentially expressed (DE) genes clusters. Clusters of DE genes were obtained by determining an optimal number of clusters using fviz_nbclust from the “factoextra” package with the gap statistic method and performing clustering using tsclust with shape-based distance (SBD), which makes the clustering particularly useful for time series where the shape matters more than exact numerical values. Clusters are standardized to log2 fold-change of abundance. Colors and cluster numbers are arbitrary. The detailed list of enriched terms is presented in Table S3. Enrichment of gene sets was performed using the Cytoscape app ClueGO with the GO biological process, KEGG pathways, and KEGG-human disease ontologies. a Clusters from stable sites. b Clusters from progressing sites. In red, metabolic activities were activated (enriched), and in blue, metabolic activities were repressed

This overall analysis does not consider the dynamic nature of the changes in gene expression. To this end, we clustered genes with the same expression pattern during our study and identified enrichment activities associated with the different gene expression clusters. In the case of human gene expression, we observed a characteristic pattern of expression in stable sites. Three different clusters were observed (Table S5). However, all of them followed the same dynamics: an increase in expression with a maximum at month 4, right before the clinical change point (Fig. 3a). The activities of these clusters are majoritarian linked to the innate and adaptive immune response (Fig. 3a). However, the progressing sites did not show signs of a robust immune response in the first months of our study. We identified 6 clusters but only 4 of them showed enrichment of GO/KEGG activities. Only cluster 1 activated the immune response, primarily when it was associated with leukocyte chemotaxis in month 2. However, it went down from that point on (Fig. 3b). Patients with chronic periodontitis show deficiencies in neutrophil chemotaxis, which may contribute to the persistence of periodontal pathogens and the progression of the disease [71]. Unlike in the stable sites in progressing sites, clusters with very different dynamics were observed (Fig. 3b, Table S5). Antigen processing and presentation never reach high activation (cluster 3), while cannabinoid receptor activity and homophilic cell adhesion presented two peaks, one before the change point (month 4) and another after the change point at month 8 (Fig. 3b). The activation of cannabinoid receptors may counteract some of these inflammatory effects, promoting a more favorable environment for periodontal healing [72]. A complete description of the enriched terms for the human expression of clusters in stable and progressing sites is presented in Table S6.

Likewise, we performed an identical analysis with the differentially expressed genes from the oral microbiome. Table S7 describes the genes that belong to the different clusters. A detailed description of the gene set enrichment results is presented in Fig. S4 (for clusters from stable sites) and Fig. S5 (clusters for progressing sites). We observed similar activities activated or suppressed when comparing stable and progressing sites. However, the times when the peaks of activation or suppression happened are very different under the two conditions. Thus, potassium transport, an activity associated with oral microbiome virulence [27, 73], peaked at month 4 in the stable sites, while the peak occurred at month 8 in progressing sites (Figs. S4 and S5). Likewise, we observed two peaks of activities associated with bacterial motility, which facilitate the penetration of periodontal pathogens through epithelial barriers, thereby exacerbating periodontal disease [74], at months 4 and 8 in progressing sites, with only an increase in stable sites at the end of the year. A similar pattern was also observed for cobalamin biosynthesis (Fig. S4 and S5), previously shown to be disease-associated [25, 27]. Galactose metabolism was inhibited at months 4 and 8 in progressing sites but at the end of the study on the stable sites. A decrease in functional genes related to glycolysis and galactose metabolism has been observed in subgingival plaques from individuals with periodontal pockets. This suggests a shift in the energy sources utilized by the microbiome in this site [75]. Similarly, tyrosine metabolism was inhibited at month 4 in progressing sites, but at the end of the study, it was on the stable sites (Fig. S4 and S5). A complete description of the enriched terms for the microbiome expression of clusters in stable and progressing sites is presented in Table S8.

### Delays of expression identify a positive feedback loop that explains the progression of the disease

Co-expression analysis can provide valuable insight into the role of molecules during biological processes but faces significant challenges in dealing with their variation in molecular response times. Timing differences or delays in initiating or suppressing molecule expression are common in biology and occur across both molecular levels and organisms. Time delay analysis finds the delay (also called the “lag”) between two signals that are shifted in time. To assess these delays in different “omics,” we used the R package DynOmics [76], a novel algorithm based on the fast Fourier transform (FFT) [76], detects, estimates, and accounts for delays between “omics” time expression profiles.

We performed the delay analysis between the profiles of expression of the host and microbiome against the progression of CAL and the delay analysis in the host-microbiome transcriptomes. Once we identified the genes with delays, we performed gene-set enrichment analysis for GO terms and KEGG pathways to identify metabolic activities with delays, either concerning the evolution of CAL with time or between host-microbiome gene expression delays.

In stable sites, the evolution of CAL is preceded only by genes associated with the FoxO signaling pathway in the host, which enhances innate immunity by regulating Toll-like receptor (TLR) signaling in macrophages and is involved in the cellular response to oxidative stress [77, 78], and no host genes had a delayed correlation with the evolution of CAL (Fig. S6a). In the microbiome, genes associated with ferric iron binding, which plays a crucial role in the pathogenesis of periodontitis [79–81], preceded the progression of CAL (Fig. S6b), while the progression of CAL preceded activities associated with protein transport by the Sec complex, cell motility, and nitrate reductase activity (Fig. S6c). Loss of beneficial bacteria with nitrate reductase capabilities may contribute to the dysbiotic state observed in the oral microbiome, leading to increased inflammation and tissue destruction [5, 82].

In the case of the progressing sites, the activation of many host activities preceded the evolution of CAL (Fig. S7a). The most significant fraction was associated with immune response, mainly response to external stimulus and leukocyte activation and migration. Only suppression of sensory perception activities preceded CAL evolution (Fig. S7a). In the case of the microbiome (Fig. S7b), we observed a high activation of activities associated with pteridine-containing compound biosynthesis, which has been highlighted as a marker of immune activation and inflammation [83, 84]. Also activated were fucose metabolism and 4 Fe- 4S clusters binding proteins, which are often present in oral pathogens and enable them to adapt to the fluctuating iron availability in the oral environment [85]. Additionally, the activation of ribosome assembly, DNA recombination, and RNA turnover point to growth or repair processes preceding the progression of CAL, possibly indicating their role in response to stress [86, 87] (Fig. S7b).

Finally, in the host, CAL preceded the activation of a different set of sensory perception activities in progressing sites (Fig. S8a), while in the microbiome, we observed an activation of porphobilinogen synthase activity involved in heme synthesis [88, 89]; spermidine metabolic process, which may contribute to protection against inflammation [90] or increased leukocyte migration [91]; and nitrate reductase activity and metabolic activities involved in carbohydrate metabolism and cofactor biosynthesis (Fig. S8b). In parallel, we observed that CAL preceded the suppression of the production of secondary metabolites, short-chain fatty acids (SCFAs) metabolism and CRISPR systems, methylation, and transcriptional regulation, highlighting the suppression of activities associated with the cell's ability to respond to stress and environmental challenges [92–94] (Fig. S8).

Additionally, we investigated the delays of expression in the host-microbiome transcriptomes. In the case of the previous analysis, all nodes (genes) were connected to only one hub (CAL), but in host-microbiome correlations, that is not the case. Given that one gene may have a delay in expression correlated with more than one single gene, this kind of analysis results in a network of delays where nodes are the host-microbiome genes with significant correlation delays.

**Fig. 4:**
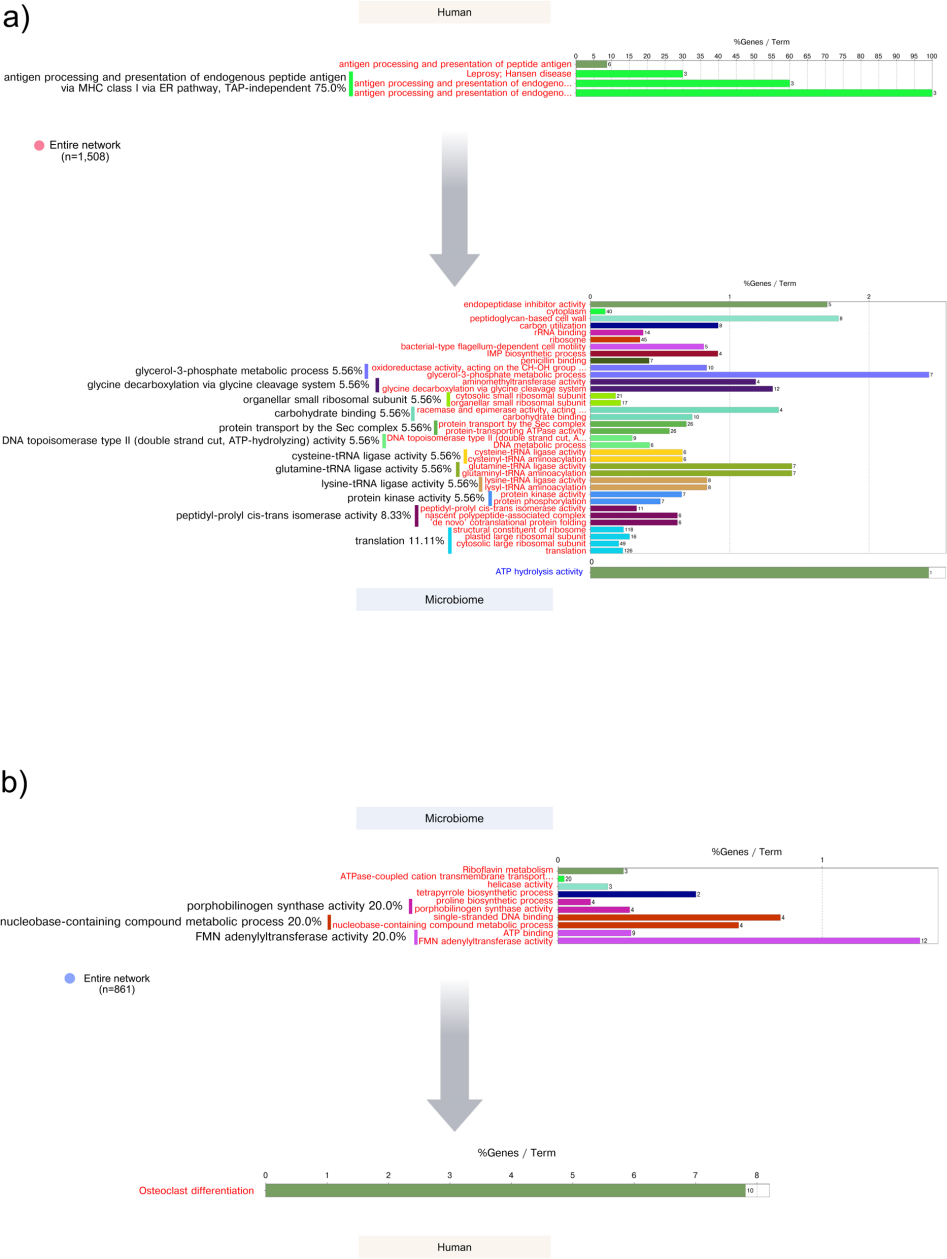
Host-microbiome delay correlation analysis, GO ontology, and KEGG pathways enrichment analysis in stable sites. Using the R package dynOmics [73], we measured the correlation between the two time series at different time lags. We identified the delay (positive or negative lag) at which the two variables most strongly correlated. a Human activities that preceded microbiome activities by 2 months. b Microbiome activities that preceded human activities by 2 months. The percentages represent the %terms/group, that is, the proportion of terms within each functional group or category relative to the total number of terms in your analysis. It shows how many enriched terms are associated with each functional group, showing their relative importance. n = number of nodes in the network. In red, metabolic activities were activated (enriched). The detailed list of enriched terms is presented in Table S7

In the case of the stable sites, activation of antigen processing and presentation activities preceded peptidyl-prolyl cis–trans isomerase activity, which has been identified as virulence factors in bacterial pathogens [95, 96], bacterial-type flagellum-dependent cell motility, and activities associated with stress responses and adaptation and protein phosphorylation (Fig. 4a, Table S9). In the case of delays of microbiome activities in relation to host activities, we observed an activation of riboflavin and tetrapyrrole metabolism are focused on producing critical cofactors (FAD, FMN, heme), proline biosynthesis, crucial for protein production and stress adaptation, and porphobilinogen synthase activity, involved in heme synthesis [88, 89]. that preceded osteoclast differentiation in the host (Fig. 4b, Table S9).

**Fig. 5:**
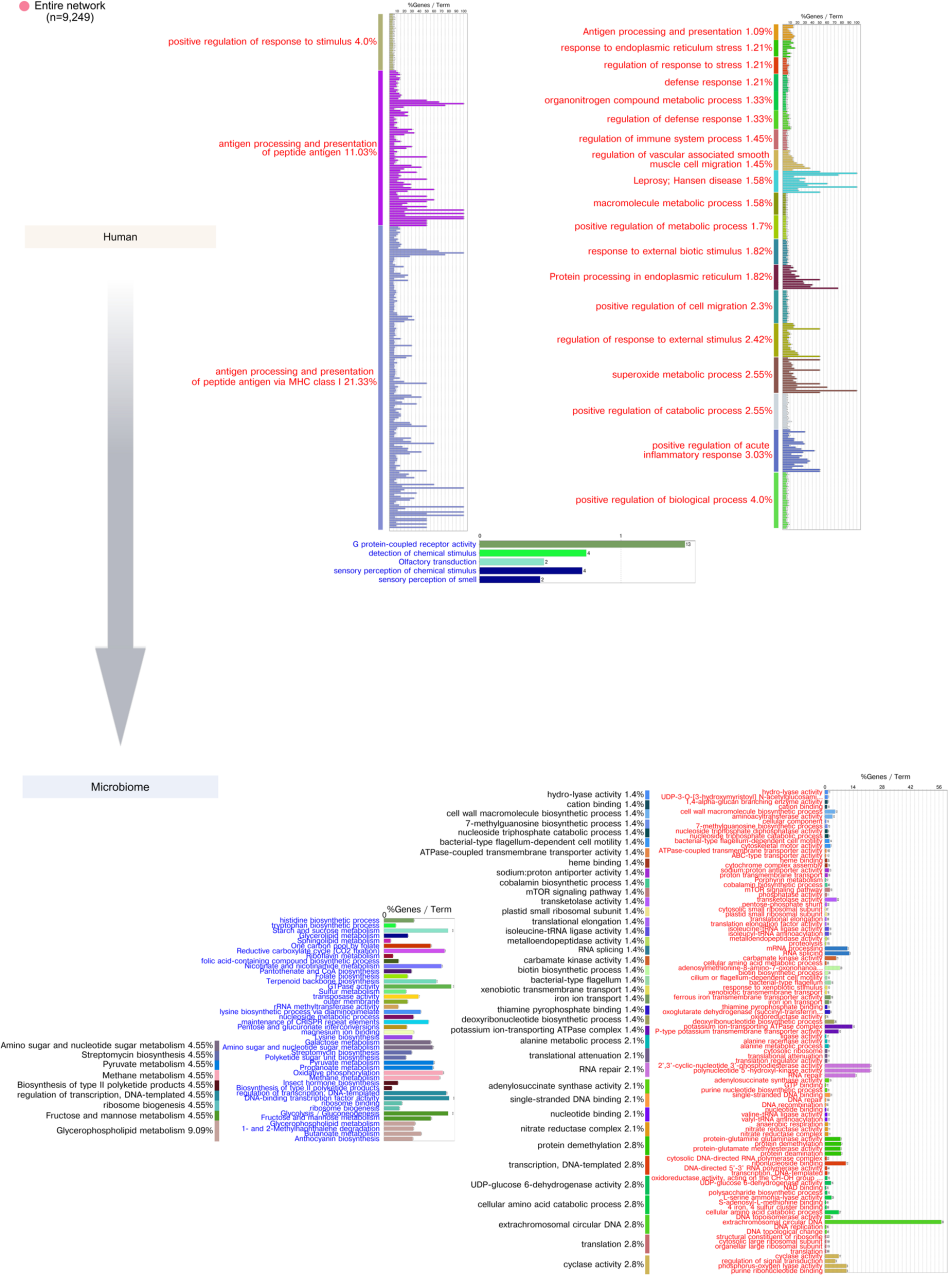
Host-microbiome delay correlation analysis and GO ontology and KEGG pathways enrichment analysis delayed gene in progressing sites. Human activities that preceded microbiome activities by 2 months. Using the R package dynOmics [73], we measured the correlation between the two time-series at different time lags. We identified the delay (positive or negative lag) at which the two variables most strongly correlated. The percentages represent the %terms/group, that is, the proportion of terms within each functional group or category relative to the total number of terms in your analysis. It shows how many enriched terms are associated with each functional group, showing their relative importance. n = number of nodes in the network. In red, metabolic activities were activated (enriched), and in blue, metabolic activities were repressed. The detailed list of enriched terms is presented in Table S8

In the progressing sites, delay analysis showed that in the host, activities associated mainly with antigen processing and presentation preceded the activation of microbiome activities associated with biosynthesis of nucleotides, cofactors, carbohydrates, iron ion transport, and heme binding, sodium: proton antiporter activity and potassium ion and xenobiotic transmembrane transport and suppression of SCFAs utilization and fatty acid metabolism, carbohydrate metabolism (amino-sugars, fructose and mannose metabolism), lipid metabolism (glycerolipids, glycerophospholipids, and sphingolipids), production of cofactors (FAD, NAD +) and precursors for vital compounds like steroids, carotenoids, and vitamins and (Fig. 5, Table S10).

**Fig. 6:**
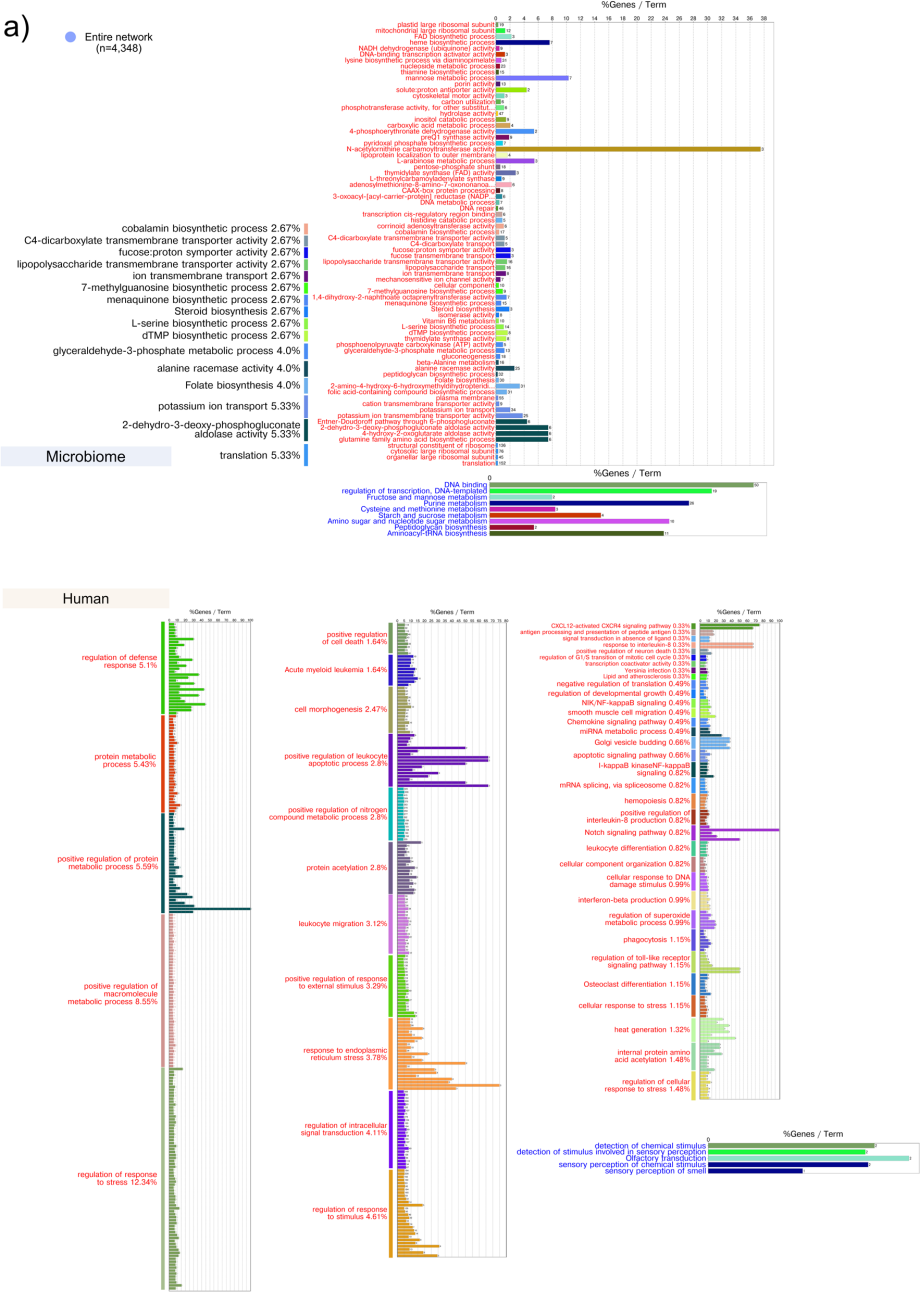
Host-microbiome delay correlation analysis and GO ontology and KEGG pathways enrichment analysis delayed gene in progressing sites. Microbiome activities that preceded human activities by 2 months. Using the R package dynOmics [73] we measured the correlation between the two time-series at different time lags. We identified the delay (positive or negative lag) at which the two variables most strongly correlated. The percentages represent the %terms/group, that is, the proportion of terms within each functional group or category relative to the total number of terms in your analysis. It shows how many enriched terms are associated with each functional group, showing their relative importance. Each group will show its contribution to the total terms identified. n = number of nodes in the network. In red, metabolic activities were activated (enriched), and in blue, metabolic activities were repressed. The detailed list of enriched terms is presented in Table S8

When we looked at microbiome activities that preceded host activities, we found that activation of biosynthesis vitamin K_2_, vitamin B_12_, steroid and folate, transport of fucose, potassium, and lipopolysaccharide and suppression of carbohydrate (amino-sugars, fructose and mannose metabolism) and amino acid metabolisms preceded activation of host activities associated with activation of immune response and defense mechanisms, apoptosis and cell death, stress response and DNA damage, NF-κB and Notch signaling pathways, and leukocyte differentiation, hemopoiesis, and migration and suppression of sensory perception of chemical stimulus (Fig. 6, Table S10).

**Fig. 7:**
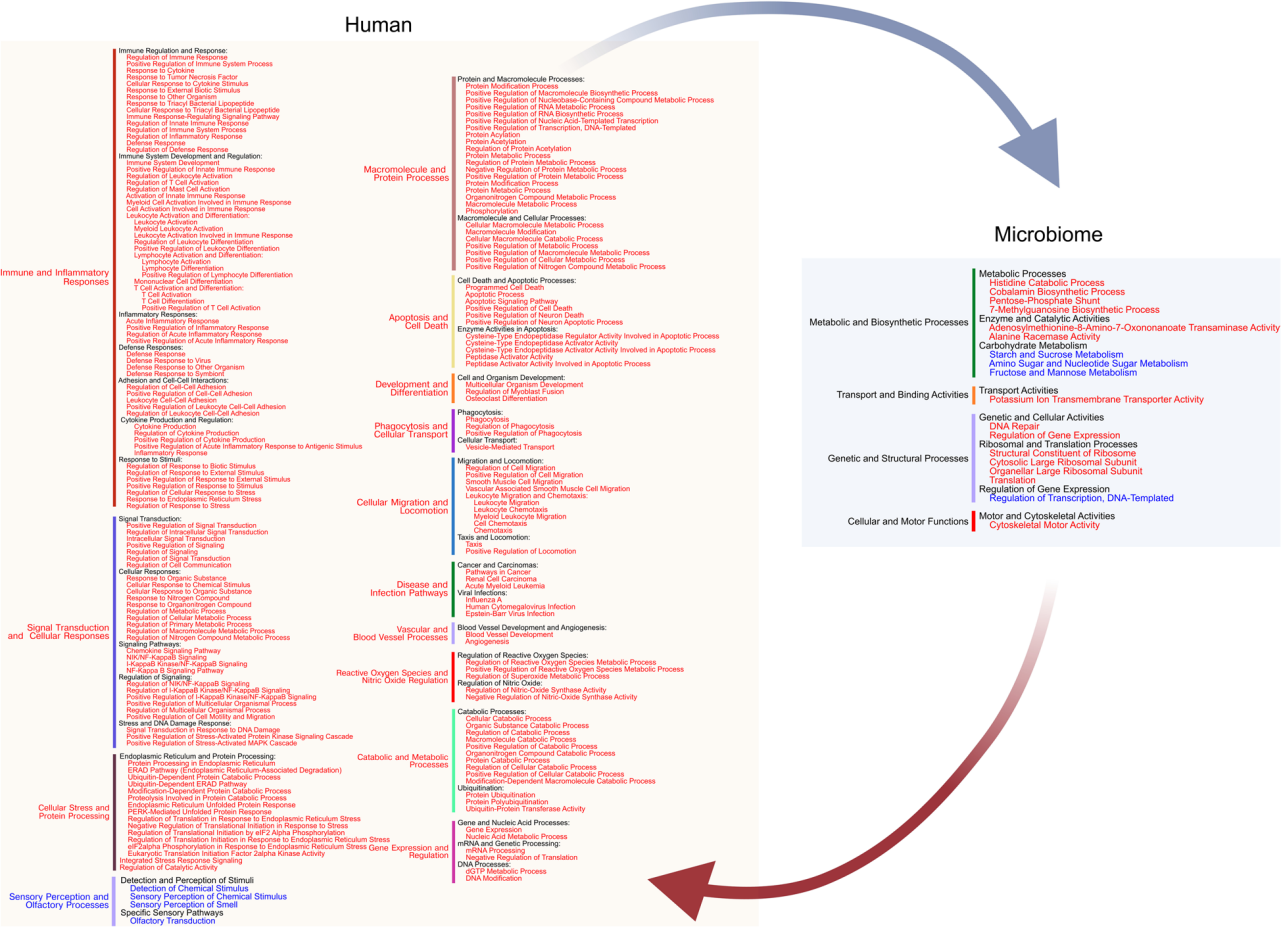
The positive feedback loop that drives the progression of the disease. Based on our delayed correlation results of host-microbiome gene expression, we found metabolic activities present in positive and negative lags. Based on these common activities, we present the infinite loop of reciprocally reinforced interactions between a dysbiotic microbiome and the host that caused the progression of the disease in red metabolic activities that were activated (enriched) and in blue metabolic activities that were repressed

Contrary to what we observed in the stable sites, certain metabolic activities from both the host and the microbiome are present in both delays, thus generating an infinite positive loop that feeds each other set of enriched activities. We have illustrated this in Fig. 7. From this kind of analysis, we cannot know what activities initiated the pathogenic loop. However, in the case of the microbiome, we found that activation of vitamin B_12_ biosynthesis, potassium transport, motility, and suppression of carbohydrate metabolism preceded activation of host activities associated with the immune response and suppression of sensory perception activities and those, in turn, preceded the previously cited microbiome activities (Fig. 7).

A focused summary of the immune response in periodontitis through gene expression data is presented in Table S11, summarizing the enrichment patterns observed across Fig. 5, 6, and 7, revealing a coordinated interaction between microbial communities and host immune responses in periodontitis. The substantial representation of microbial metabolic pathways (particularly amino sugar metabolism and glycerophospholipid metabolism) alongside host immune activation suggests an active microbial influence on disease pathogenesis. Simultaneously, the high enrichment of MHC class I presentation pathways alongside inflammatory regulatory mechanisms indicates a complex host response that balances protective immunity and destructive inflammation in periodontitis lesions.

### The trajectories of expression of three gene clusters determine the outcome of the disease

**Fig. 8:**
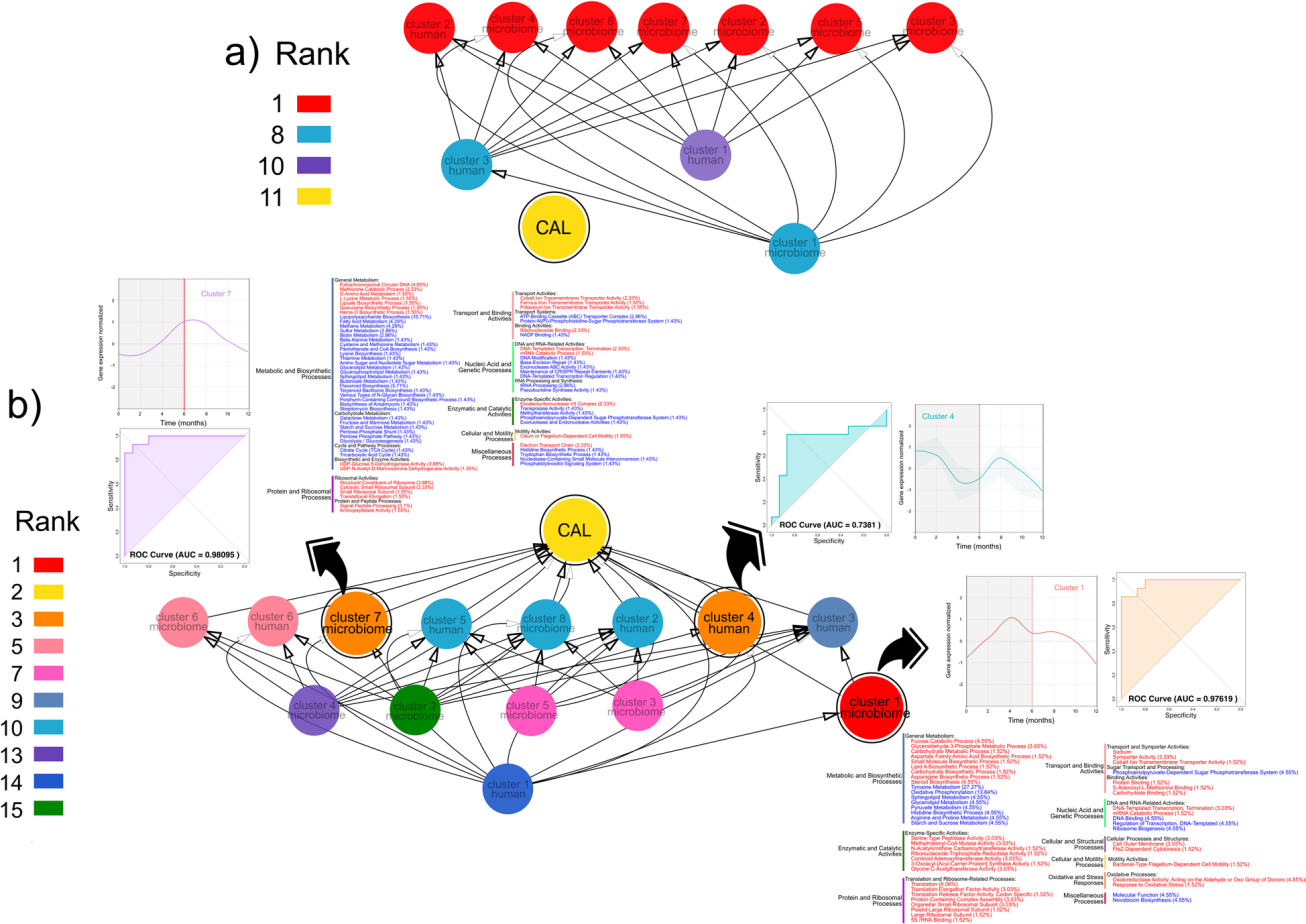
Causal linkages between host-microbiome activities and progression of CAL using transfer entropy (TE) analysis. We used the clusters of DE genes and the progression of CAL as the time series to measure TE. We calculated all pairwise causal relationships between the different clusters and CAL. The importance of the nodes as essential elements in a network was measured using the density of maximum neighborhood component (DMNC) algorithm from “cytoHubba,” which gave us the ranks for the different nodes. Significant clusters were modeled using recurrent neural networks (RNN), and ROC curves were calculated to assess their performance. In red, metabolic activities were activated (enriched), and in blue, metabolic activities were repressed. a Expression clusters from stable sites. b Expression clusters from progressing sites

Next, we investigated potential causal linkages between host-microbiome activities and clinical phenotypes (progression of CAL) using transfer entropy (TE) analysis. In the previous section, we studied the dynamics of delay correlations, but they do not necessarily imply causality. TE is a powerful tool for assessing causal relationships in complex systems, particularly when dealing with non-linear interactions and multivariate time series data [97–99]. To identify the activities that forecast the evolution of CAL and, therefore, the disease’s progression, we used the R package *NlinTS* [100] to calculate TE as a causality measure. We used the cluster of DE genes identified above as the time series to identify the causality of CAL. We calculated all pairwise causal relationships between the different clusters and CAL. Thus, networks of causality were generated from those results (Fig. 8). To identify hub nodes or essential elements in a network, we used the density of maximum neighborhood component (DMNC) algorithm from “*cytoHubba*” [101]. DMNC is a significant metric in network analysis, particularly within biological networks [102, 103]. DMNC is utilized to assess the connectivity and importance of nodes in complex interactomes, providing insights into the functional roles of various proteins and genes in biological systems. Based on the DMNC results, “cytoHubba” ranks the networks by importance. In the case of the stable sites, none of the clusters of DE genes were connected to the evolution of CAL during the study (Fig. 8a). In fact, CAL was the node with the lowest rank in importance in the network. However, in the case of progressing sites, 3 clusters of DE genes, two from the microbiome and one from the host, forecasted the progression of CAL and ranked among the top 3 nodes in the network (Fig. 8b). In fact, cluster 1 from the microbiome ranked higher than CAL on the DMNC ranks for the network nodes.

Cluster 1 from the microbiome ranked first and peaked before the change point. The genes were associated with the activation of steroid biosynthesis, carbohydrate metabolism, peptidase activity, ion transport, bacterial motility, and response to oxidative stress and suppression of lipid metabolism (Fig. 8b). Cluster 7 from the microbiome had a peak around the change point and was also associated with activation of ion transport (cobalt, iron, and potassium), heme synthesis, peptidase activity and cell motility and suppression of lipid metabolism, porphyrin-containing compounds and terpenoid biosynthesis, carbohydrate metabolism and DNA repair (Fig. 8b). The expression of cobalt uptake transport proteins is vital for the de novo biosynthesis of vitamin B_12_ in *Escherichia coli* [104]. Finally, cluster 4 from human DE genes, ranked third like cluster 7 from the microbiome, peaked at the beginning and after the change point. However, when we performed gene set enrichment analysis on the genes from cluster 4 of the host, we found no enriched terms.

In our final assessment of the validity of this cluster to predict the progression of CAL (stable or progressing), we used recurrent neural networks (RNN) to model them. RNNs are a class of robust neural networks for modeling sequence data such as time series [105, 106]. We performed our analysis in R using “*keras*” and *“tensorflow*” [107]. The results are presented in Fig. 8b as ROC curves for each cluster. Although the 3 clusters performed well in forecasting the outcome of a site, the two microbiome clusters had a better AUC (cluster 1 AUC = 0.97619 and cluster 7 AUC = 0.98095) than the host cluster (cluster 4 AUC = 0.7381).

## Discussion

Integrating metatranscriptomic data with traditional metagenomic analyses enhances our understanding of the oral microbiome’s role in health and disease. As highlighted by Huang et al., combining these methodologies allows for comprehensive profiling of microbial communities, linking specific functional activities to disease states such as periodontitis and systemic conditions [108]. Although most studies are based on associations it has been established that there is a link between periodontal dysbiosis and conditions such as cardiovascular diseases [109, 110], diabetes [111, 112], and even certain cancers [113–115]. Poor periodontal health has been associated with increased serum levels of inflammatory mediators, which can exacerbate systemic inflammatory diseases [4, 116]. Furthermore, the inflammatory mediators released during periodontal infections may enter systemic circulation, influencing atherosclerosis, chronic obstructive pulmonary disease (COPD), and rheumatoid arthritis [117, 118]. Moreover, the interaction between periodontal pathogens and gut microbiota reinforces the systemic implications of oral dysbiosis. Studies reveal that oral administration of *P. gingivalis* can induce gut microbiota dysbiosis, leading to compromised gut barrier function and potential endotoxemia [119, 120].

To understand the contribution of the oral microbial community to periodontitis progression, we must investigate the dynamics of the community in progression and stability. It is crucial to note that the majority of studies are currently cross-sectional. This approach only provides a snapshot of a highly variable microbiome at a specific time, which may not accurately represent an individual’s average composition or composition at a relevant moment in the periodontitis development timeline. This limitation underscores the urgent need for more comprehensive and longitudinal studies. In the present study, we followed a cohort of 15 patients for a period of 1 year; samples were taken every 2 months, and the host-microbiome metatranscriptome was on stable and progressing sites. Based on these results, we identify a set of principles that describe the reciprocally reinforced interactions between a dysbiotic microbiome and the host inflammatory response that lead to the progression of the disease.

For over a century, the central dogma of periodontal disease has been tissue destruction resulting from a hyper-inflammatory response to a dysbiotic microbiome [30, 121]. The traditional belief in microbiology has been that virulence is an inherent property of certain organisms, allowing them to cause disease. However, we now understand disease within an ecological framework, where virulence emerges from the interactions between hosts and pathogens and their relationship with the environment rather than being solely determined by the microbes or the host [122, 123]. In the case of periodontal disease, we now appreciate that there is a “commensal-to-pathogen” spectrum. Thus, under specific changes in the environment, some species can act as accessory pathogens, enhancing the colonization or metabolic activity of pathogens [124]. In contrast, others considered commensal, such as *Streptococcus* species, can synthesize a large number of putative virulence factors [25, 27, 73]. As a part of this “commensal-to-pathogen” spectrum, certain low-abundance microbial pathogens, defined as “keystone pathogens,” can orchestrate inflammatory disease by remodeling a usually benign microbiota into a dysbiotic [125–127].

One critical variable in the time series analysis is the change point, which is the point where an abrupt change occurs in chronologically ordered observations. change point analysis has been used in various scientific fields [128–130], but in biology, its use has been primarily focused on epidemiological studies, such as the modeling of disease outbreaks [131–133]. Our study uses this technique to identify the temporal change point at which the sites were clinically divergent. This change point would serve as the reference to search for metatranscriptome changes that may be critical to the progression of the disease. In our patients, the change point coincided with the 6-month time point in the progressing sites. Moreover, we observed drastic changes during this initial period that would define the clinical outcome of specific sites.

Previous metagenomic analysis of clinical disease progression showed that changes in microbial community composition followed an ecological succession, and temporal fluctuations of the different bacterial clusters were observed, with peaks and valleys of abundance. Moreover, in some cases, two or more clusters shared temporal peaks but behaved differently at other times [134]. We observed similar behavior in our metatranscriptome analysis, where transcripts were phylogenetically assigned to different species as a proxy to active communities. We identified clusters of active bacteria that followed different profiles in stable and progressing sites. Interestingly, the peak of *P. gingivalis* and *T. forsythia*, two important periodontopathogens [20, 61], occurred at the change point, while in stable sites, they never reached high abundance. On the other hand, *F. nucleatum* and *Leptotrichia buccalis*, two organisms that have been associated with gingivitis, the initial stages of periodontitis [20, 31] showed a peak of activity at month 4 before the change point, probably contributing to the establishment of an environment favorable to disease progression.

Our major interest was to characterize host-microbiome functional activities that define and predict the progression of the disease. Previous cross-sectional metatranscriptomic studies assessed changes in progressing and non-progressing subgingival tooth sites. They showed that oral microbial communities in progressing sites are metabolically distinct from those in non-progressing sites and that the differences are detectable prior to disease progression. The progressing sites displayed enrichment of pathogenesis-associated functional signatures, including oxidative stress response, amino acid transport, ferrous iron transport, lipid A biosynthesis, cell motility [25, 27], and impaired carbohydrate metabolism [135].

Here, we demonstrated that stable sites mounted a robust immune response before the change point, especially regarding acquired immunity with activation of antigen processing and presentation and T cell proliferation that is not present in progressing sites. Previous studies have shown that T-cells extracted from diseased periodontal tissues exhibit a reduced response to stimuli, which suggests that the cell-mediated response is suppressed in patients with periodontal disease [136]. Simultaneously, we observed a suppression of activities associated with sensory perception in stable sites. Gingival solitary chemosensory cells (SCC) in the sulcular and junctional epithelium are epithelial sentinels that use bitter taste receptors to detect bacterial metabolites evoking innate immune responses and the release of antimicrobial compounds, thus shaping the composition of the microbiome [137, 138].

The host response in progressing sites was defined by an initial effort to mount an immune response that vanished with time and two peaks of activation of activities associated with cannabinoid receptor activity, one before and one after the change point. The endocannabinoid system (ECS) plays a significant role in the periodontal healing. Cannabinoids, particularly through the activation of CB2 receptors, can promote the proliferation of gingival fibroblasts, which are crucial for tissue regeneration during periodontal healing [139, 140]. Studies have shown that selective CB2 receptor agonists can mitigate lipopolysaccharide (LPS)-induced inflammation in periodontal tissues, suggesting that modulation of the ECS may provide a therapeutic avenue for controlling periodontal inflammation [139, 141, 142]. Furthermore, the presence of endocannabinoids such as anandamide (AEA) and 2-arachidonoylglycerol (2-AG) in gingival crevicular fluid (GCF) of individuals with periodontal disease indicates their involvement in the inflammatory response associated with periodontitis [143, 144]. In the case of the metatranscriptome of the oral microbiome, we found that several activated and suppressed activities were common in stable and progressing sites. In this case, rather than activating or suppressing specific metabolic activities in the microbiome, a specific sequence of events that would lead to progression or stability seems critical for the outcome of a specific site. For example, galactose and tyrosine metabolism are suppressed at the end of the study in stable sites but before and after the change point. Galactose metabolism in oral pathogens, specifically *F. nucleatum* and *T. forsythia*, involves complex interactions contributing to their pathogenicity and metabolic pathways.. Recent studies have indicated that galactose can disrupt the biofilm formation of periodontal pathogens, suggesting that it may serve as a signaling molecule affecting microbial interactions in the oral cavity [145, 146]. *F. nucleatum*, a key player in oral biofilms, expresses Fap2, a galactose-binding lectin critical for its adhesion to host tissues and other bacterial species. The Fap2 adhesin is known to facilitate coaggregation with *P. gingivalis* and may play a vital role in periodontal disease pathogenesis by allowing these bacteria to colonize and thrive in biofilms within the oral cavity [147, 148]. The binding of Fap2 to galactose is crucial for its adhesion processes, enhancing the survival of *F. nucleatum* in competitive microbial ecosystems [148]. Moreover, its ability to aggregate with other periodontal pathogens, including *P. gingivalis* and *T. forsythia*, amplifies the pathogenic potential of *F. nucleatum* by fostering synergistic relationships that contribute to periodontal disease severity [149]. *T. forsythia* similarly exhibits metabolic competencies, with studies suggesting that its ability to metabolize different carbohydrates is key to its role in oral biofilms alongside *P. gingivalis* and *F. nucleatum* [150, 151]. Furthermore, the presence of galactose may enhance the virulence of *F. nucleatum*. It is well-documented that galactose and related carbohydrates can affect interactions between bacteria and host cells, influencing inflammatory responses and potentially leading to chronic conditions such as periodontitis [152].

Similarly, potassium transmembrane transporter activity and ornithine metabolic processes were activated at month 4 in stable sites but at month 8 in progressing sites. Potassium transport has been linked to increased virulence in oral bacteria [73]. Ornithine plays a significant role in the pathogenesis of periodontitis, particularly through the interactions of various oral bacteria and their metabolic byproducts [153, 154], and elevated levels of ornithine have been observed in the gingival crevicular fluid of periodontitis patients, suggesting that it may serve as a biomarker for disease severity [155]. Studies have shown that the oral commensal *Streptococcus gordonii* can release ornithine, which subsequently supports the growth of *Fusobacterium nucleatum*, a key pathogen in periodontal disease [156, 157]. This metabolic cooperation underscores the complex interspecies interactions within the periodontal biofilm, where certain bacteria can significantly alter the local metabolic environment.

Other activities were thought to be associated with one of the two clinical conditions. Thus, histidine biosynthesis was suppressed at month 4, and its degradation was activated at months 4 and 5 in progressing sites but not altered in stable sites. In a study by Jorth et al., the authors found enhanced gene expression in all diseased sites of three patients for histidine catabolism [28].

As briefly discussed above, periodontitis is driven by reciprocally reinforced interactions between a polymicrobial dysbiotic community and an exaggerated host inflammatory response, leading to a cycle of inflammation and tissue degradation that perpetuates the disease process [30, 121]. The nature of the microbial activities contributing to this positive feedback loop is largely unknown [158]. Applying delay correlation analysis to our data, we identified activities in the host and the microbiome that preceded each other, thus explaining why this positive feedback loop does not stop unless either dysbiosis or inflammation is targeted. Activated host activities were mainly associated with immune response, inflammation, apoptosis, and cell adhesion, while suppressed activities were associated with sensory pathways. In the microbiome, activated activities of the feed-forward loop were associated with histidine catabolic processes, cobalamin biosynthesis, potassium transport, pentose-phosphate shunt, 7-methylguanosine biosynthetic process, adenosylmethionine- 8-amino- 7-oxononanoate transaminase activity, alanine racemase activity and DNA repair and suppressed activities were associated with sugar metabolism. Additionally, we observed that in progressing sites CAL and activities associated with antigen processing and presentation preceded suppression of the production of short-chain fatty acids (SCFAs) in the microbiome. There is increase evidence of the complex interplay between diet, gut microbiota and SCFA production, collectively shaping the host's inflammatory and metabolic responses [159, 160]. In the intestine SCFAs exert protective effects against inflammation and contribute to beneficial metabolic functions through their actions on immune cell signaling and energy regulation They can modulate immune responses by activating specific G protein-coupled receptors (GPCRs) expressed on immune cells and epithelial cells of the gut,such as GPR41 and GPR43, which are, facilitating the resolution of inflammation through the modulation of cytokine production and the promotion of anti-inflammatory pathways [161, 162]. Thus butyrate can inhibit the activation of pro-inflammatory pathways and enhance the production of anti-inflammatory cytokines [163, 164]. SCFAs have been associated with maintaining intestinal barrier function, reducing mucosal inflammation, and promoting gut homeostasis [165] and the gut microbiota’s ability to produce SCFAs is closely tied to the host's susceptibility to metabolic disorders, including obesity and type 2 diabetes [166].

Our metatranscriptomic analysis also revealed suppression of activities associated with amino-sugar metabolism and glycan degradation pathways, particularly in progressing sites. These sites exhibit increased expression of enzymes targeting glycosphingolipids and glycoproteins, which are integral components of host-derived glycoconjugates. Sialic acids are a critical nutrient for the oral periodontal pathogen *T. forsythia*. *T. forsythia* has adapted mechanisms to exploit sialic acid, an abundant component in the oral cavity, particularly in glycoproteins in saliva and gingival crevicular fluid. This sialic acid can be utilized by *T. forsythia*, promoting its growth and biofilm formation [167–169]. *T. forsythia* utilizes the enzyme sialidase, which hydrolyzes sialic acid residues, enhancing its ability to scavenge and metabolize these sugars for energy, effectively stimulating biofilm growth [168, 169]. *P. gingivalis* also exhibits substantial sialidase activity, which modulates its virulence by altering glycoproteins in the host environment. The enzymatic activity of sialidases in this bacterium enhances the proteolytic activity of other virulence factors, such as gingipains, thus facilitating tissue degradation and inflammation [153, 170]. This activity may contribute to microbial persistence and immune modulation in the subgingival environment.

In the context of microbiome research, Sazal et al. emphasized the shift from associative to causal inference, which is crucial for understanding complex biological processes [171]. This transition is vital for accurately interpreting the interactions within microbial communities and their effects on host biology. We used transfer entropy to assess causal inference of CAL on clusters of differentially expressed genes. Transfer entropy is a powerful tool for assessing causal relationships in time series data, particularly in short time series and non-linear systems where traditional methods may struggle due to limited data points [172]. No cluster of genes showed causality with the evolution of CAL in stable samples. However, in the progressing sites, 3 clusters of differentially expressed genes, two from the microbiome and one from the host, were able to infer the outcome of progressing sites. We modeled the ability of these clusters to discriminate between stable and progressing sites, and the AUC of the host cluster was lower than the AUC of the two microbiome clusters, which had excellent discrimination ability.

## Conclusion

**Fig. 9:**
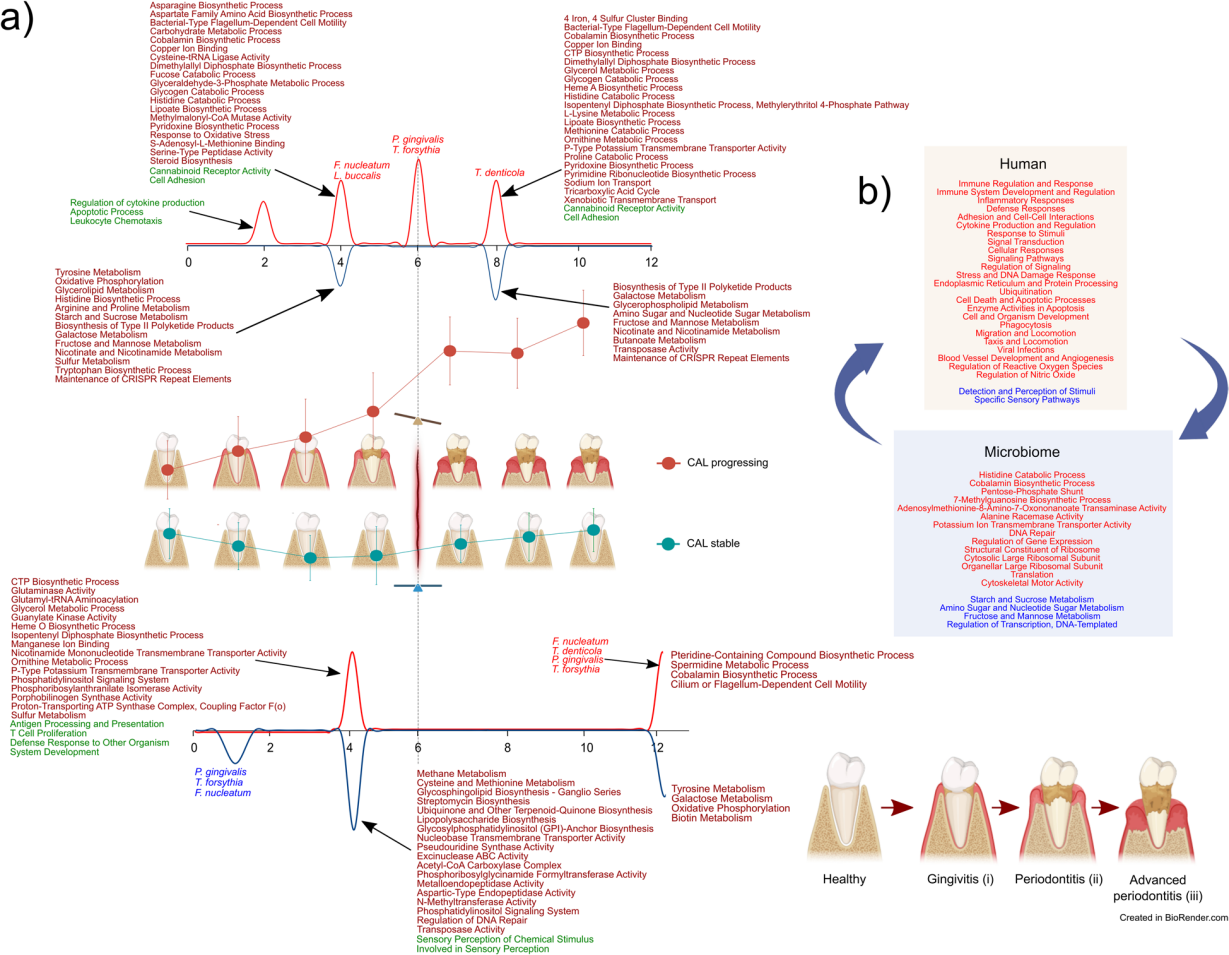
Periodontitis progression model comparing temporal hallmarks in stable and progressing sites. a Timeline of host-microbiome activities in stable and progression sites. b Host-microbiome activities associated with the positive feedback loop leading to progression

Our study established a timeline of host-microbiome activities that define the site outcome in periodontitis patients (Fig. 9a). Moreover, we identified the activities involved in the positive feed-forward loop that may explain progression why, once the disease starts, it progresses until the tooth is lost unless there is clinical intervention (Fig. 9b). Finally, based on the profiles of DE genes, we elucidated the causal relationships between these gene clusters and CAL in progressing sites, pointing out microbial activities that can forecast the evolution of a site.

## Supplementary Information

Additional file 1. Figure S1.Experimental design. 15 participants were selected from a total cohort of 415 participants. These patients had the clinal conditions we wanted: progressing sites where CAL increased steadily and significantly during the study and stable sites where CAL values remained significantly identical. Genetic background should have a minimal effect on the outcome of individual sites. At baseline, all teeth used were clinically identical. Subgingival plaque samples were taken every 2 months for 1 year, after which all patients underwent scaling and root planing as treatment. After 3 months for a visual check-up and again after 6 months when they were monitored, all returned to the clinic, and samples were also taken. Figure S2. Phylogenetic assignment and relative quantification of microbiome metatranscriptome. a) Combined clusters with peaks before the change point. b) Venn diagram of the combined species from the stable and progressing clusters. c) List of the species in the three sections of the Venn diagram. Figure S3. Gene set enrichment analysis of the overall changes in the study. We performed gene set enrichment analysis of GO terms and KEGG pathways using *clusterProfiler* [49] on the DE from the host and the microbiome. In red activated enriched activities. In green suppressed enriched activities. Figure S4. Microbiome GO ontology and KEGG pathways enrichment analysis of differentially expressed (DE) gene clusters in stable sites. Clusters of DE genes were obtained by determining an optimal number of clusters using *fviz_nbclust* from the ‘*factoextra*’ package with the gap statistic method and performing clustering using *tsclust* with shape-based distance (SBD), which makes the clustering particularly useful for time series where the shape matters more than exact numerical values. Clusters are standardized to log2 fold-change of abundance. Colors and cluster numbers are arbitrary. The actual composition of the different clusters is presented in Table S2. Enrichment of gene sets was performed using the Cytoscape app ClueGO with the GO biological process, KEGG pathways, and KEGG-human disease ontologies. In red, metabolic activities were activated (enriched), and in blue, metabolic activities were repressed. Figure S5. Microbiome GO ontology and KEGG pathways enrichment analysis of differentially expressed (DE) gene clusters in progressing sites. Clusters of DE genes were obtained by determining an optimal number of clusters using *fviz_nbclust* from the ‘factoextra’ package with the gap statistic method and performing clustering using *tsclust* with shape-based distance (SBD), which makes the clustering particularly useful for time series where the shape matters more than exact numerical values. Clusters are standardized to log2 fold-change of abundance. Colors and cluster numbers are arbitrary. The actual composition of the different clusters is presented in Table S5. Enrichment of gene sets was performed using the Cytoscape app ClueGO with the GO biological process, KEGG pathways, and KEGG-human disease ontologies. In red, metabolic activities were activated (enriched), and in blue, metabolic activities were repressed. Figure S6. CAL-host and microbiome delay correlation analysis, GO ontology, and KEGG pathways enrichment analysis in stable sites. Using the R package dynOmics [73] we measured the correlation between the two time-series (CAL and host or microbiome genes) at different time lags. We identified the delay (positive or negative lag) at which two variables correlate most strongly. a) CAL profile preceded human activities by 2 months. b) Microbiome activities that preceded CAL by 2 months. c) CAL profile preceded microbiome activities by 2 months. The percentages represent the %terms/group, that is, the proportion of terms within each functional group or category relative to the total number of terms in your analysis. It shows how many enriched terms are associated with each functional group, showing their relative importance. n = number of nodes in the network. In red, metabolic activities were activated (enriched), and in blue, suppressed activities were suppressed. Figure S7. Host and microbiome-CAL delay correlation analysis, GO ontology, and KEGG pathways enrichment analysis in progressing sites. Using the R package dynOmics [73] we measured the correlation between the two time-series (CAL and host or microbiome genes) at different time lags. We identified the delay (positive or negative lag) at which two variables correlate most strongly. a) Human activities that preceded CAL by 2 months. b) Microbiome activities that preceded CAL by 2 months. The percentages represent the %terms/group, that is, the proportion of terms within each functional group or category relative to the total number of terms in your analysis. It shows how many enriched terms are associated with each functional group, showing their relative importance. n = number of nodes in the network. In red, metabolic activities were activated (enriched), and in blue, suppressed activities were suppressed. Figure S8. CAL-host and microbiome delay correlation analysis, GO ontology, and KEGG pathways enrichment analysis in progressing sites. Using the R package dynOmics [73] we measured the correlation between the two time-series (CAL and host or microbiome genes) at different time lags. We identified the delay (positive or negative lag) at which two variables correlate most strongly. a) CAL profile preceded human activities by 2 months. b) CAL profile preceded microbiome activities by 2 months. The percentages represent the %terms/group, that is, the proportion of terms within each functional group or category relative to the total number of terms in your analysis. It shows how many enriched terms are associated with each functional group, showing their relative importance. n = number of nodes in the network. In red, metabolic activities were activated (enriched), and in blue, suppressed activities were suppressed.

Additional file 2.

Additional file 3.

Additional file 4.

Additional file 5.

Additional file 6.

Additional file 7.

Additional file 8.

Additional file 9.

Additional file 10.

Additional file 11.

Additional file 12.

Additional file 14.

## Data Availability

The datasets generated and/or analyzed during the current study are available in the Sequence Read Archive (SRA) data repository of NCBI with BioProject ID PRJNA770647 (https://www.ncbi.nlm.nih.gov/sra/?term = PRJNA770647). In addition, all code and software used to process and analyze the data are available as a fully reproducible computing environment in the jupyter notebook provided in the supplementary materials.
