# Correction: Longitudinal host-microbiome dynamics of metatranscription identify hallmarks of progression in periodontitis

**DOI:** 10.1186/s40168-025-02170-2

**Published:** 2025-06-20

**Authors:** Ana Duran‑Pinedo, Jose O. Solbiati, Flavia Teles, Zhang Yanping, Jorge Frias‑Lopez

**Affiliations:** 1 Department of Oral Biology, University of Florida, College of Dentistry, 1395 Center Drive Gainesville, Gainesville, FL 32610 ‑ 0424 USA; 2 Department of Basic & Translational Sciences, University of Pennsylvania, School of Dental Medicine, 240 South 40 Street, Philadelphia, PA 19104 ‑ 6030 USA; 3 Center for Innovation and Precision Dentistry (CiPD), University of Pennsylvania, School of Dental Medicine, 240 South 40 Street, Philadelphia, PA 19104 ‑ 6030 USA; 4 Gene Expression & Genotyping Core, Interdisciplinary Center for Biotechnology Research, University of Florida, 178 B CGRC, 2033 Mowry Road, Gainesville, FL 32610 USA

**Correction: Microbiome 13, 119 (2025)**

**https://doi.org/10.1186/s40168-025-02108-8**

Following publication of the original article [1], the authors identified an error in the name of all authors. The given name and family name were erroneously transposed.

The incorrect author names are: Duran‑Pinedo Ana, Solbiati Jose O, Teles Flavia, Yanping Zhang and Frias‑Lopez Jorge.

The correct author names are: Ana Duran‑Pinedo, Jose O Solbiati, Flavia Teles, Zhang Yanping and Jorge Frias‑Lopez.

The author group has been updated above and the original article [1] has been corrected.
