# Supplementary material for: Longitudinal host-microbiome dynamics of metatranscription identify hallmarks of progression in periodontitis
Source: Microbiome. 2025 May 14;13:119. doi: 10.1186/s40168-025-02108-8 (PMC12077055; doi:10.1186/s40168-025-02108-8)
Supplement: Supplementary file 4 — Additional file 3. [file 40168_2025_2108_MOESM3_ESM.docx]

**Immunological Signatures in Periodontitis: Interpretive Guide for Figures 5-7**

This supplemental guide highlights key immune-related pathways from our comprehensive enrichment analysis in Figures 5-7, focusing on immunological signatures that connect microbial changes with host responses in periodontitis.

**Summary Microbiome-Host Interactions in Figure 6**

**Human**

- Antigen Processing and Presentation Mechanisms

- Antigen processing and presentation of peptide antigen via MHC class I (21.33%) and antigen processing and presentation (11.66%): These highly enriched pathways demonstrate activation of immune adaptive response. The substantial enrichment in MHC class I presentation suggests strong cytotoxic immune surveillance in periodontitis lesions, while the overall antigen presentation enrichment indicates broad immune system engagement with periodontal pathogens.

- Inflammatory Response Regulation

- Positive regulation of acute inflammatory response (3.03%) and positive regulation of biological process (4.0%): These pathways reflect the active inflammatory state characteristic of periodontitis. Their enrichment demonstrates the host's coordinated response to microbial challenges in the periodontal pocket.

- Defense and Stress Responses

- Defense response (1.21%), response to stress (1.21%), and response to external biotic stimulus (1.82%): This cluster of stress-response pathways, while moderately enriched, represents the activation of protective mechanisms against microbial challenge.

**Microbiome**

- Amino sugar and nucleotide sugar metabolism (4.55%) and Glycerophospholipid metabolism (9.09%): These prominent microbial metabolic pathways suggest active bacterial metabolism that may influence the local periodontal environment and host-microbe interactions.
- Cell wall macromolecule biosynthetic process (1.4%) and bacterial-type flagellum-dependent cell motility (1.4%): These bacterial structural components are known to be immunostimulatory, potentially serving as pathogen-associated molecular patterns (PAMPs) that trigger host immune recognition via toll-like receptors.
- Hydrocarbon oxygenase activity (1.4%) and glycosyltransferase activity (1.4%): These enzymatic activities in the microbiome may contribute to biofilm formation and matrix modification, potentially affecting bacterial persistence in periodontal pockets.

**Summary Microbiome-Host Interactions in Figure 6**

**Human**

- Immune Cell Function and Trafficking

- Leukocyte migration (2.12%) and positive regulation of cell death (7.84%): These pathways highlight key mechanisms of the destructive inflammatory process in periodontitis. Leukocyte recruitment coordinates with programmed cell death processes, potentially explaining tissue destruction observed clinically.

- Inflammatory Signaling

- Regulation of NF-κB signaling (1.12%) and positive regulation of response to external stimulus (1.99%): These regulatory pathways orchestrate the inflammatory cascade in response to microbial stimuli, representing central control mechanisms in periodontal inflammation.

**Microbiome**

- Translation (5.33%) and potassium ion transport (5.33%): These prominent microbial activities may influence local microenvironment conditions and produce immune-stimulating virulence factors.

**Integrated Immunopathological Networks in Figure 7**

- Major Immune Component Clusters

- Immune Regulation and Response and Inflammatory Responses: These extensive networks at the left side of Figure 7 illustrate the central role of coordinated immune activation in periodontitis pathogenesis.

- Cellular Response Mechanisms

- Signal Transduction and Cellular Responses and Cellular Stress and Protein Processing: These interconnected networks reveal how host cells detect and respond to microbial challenges, linking receptor activation to effector responses.

- Metabolic-Immune Interface

- Catabolic and Metabolic Processes and Reactive Oxygen Species Regulation: These pathways demonstrate the importance of oxidative stress components that contribute to tissue damage.
