# Supplementary material for: Longitudinal host-microbiome dynamics of metatranscription identify hallmarks of progression in periodontitis
Source: Microbiome. 2025 May 14;13:119. doi: 10.1186/s40168-025-02108-8 (PMC12077055; doi:10.1186/s40168-025-02108-8)
Supplement: Supplementary file 5 — Additional file 4. [file 40168_2025_2108_MOESM4_ESM.pdf]

Figure S1

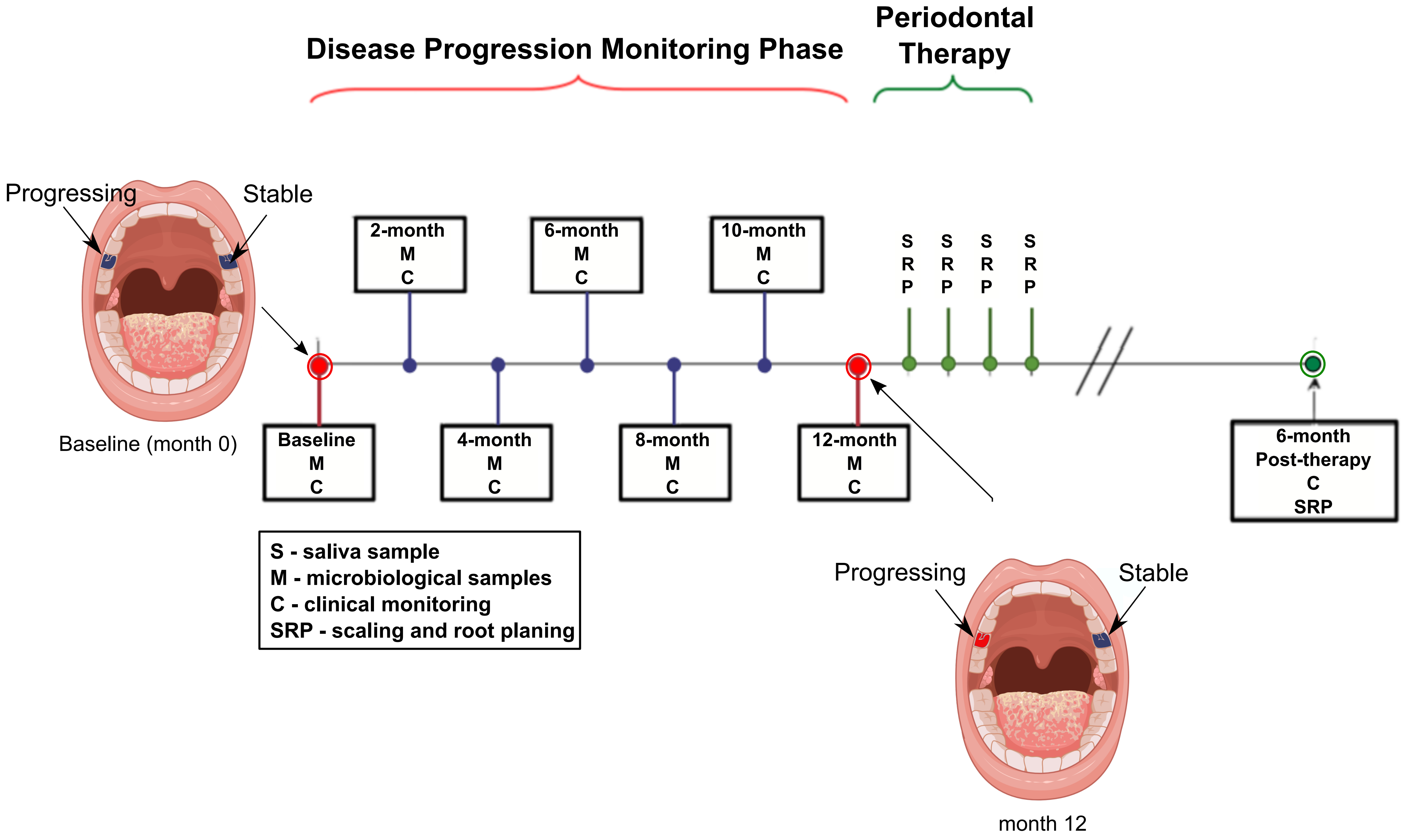

Created in BioRender.com

a) b)

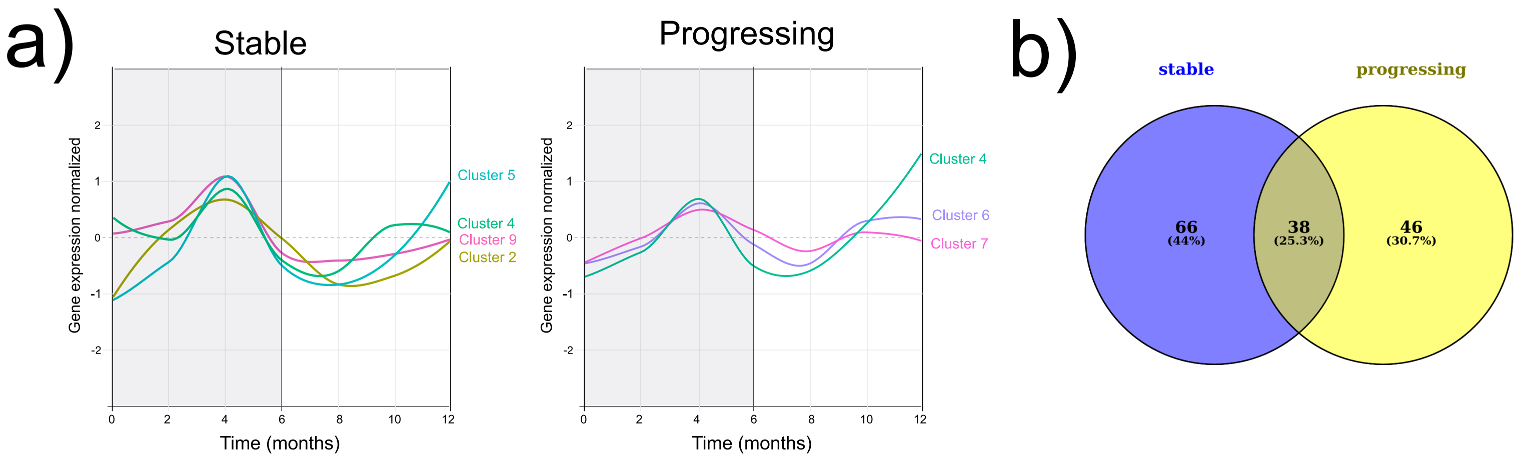

c)

Exclusively in stable

Actinomyces_naeslundii
Actinomyces_oris
Actinomyces_sp._oral_taxon_170
Actinomyces_sp._oral_taxon_175
Actinomyces_sp._oral_taxon_180
Actinomyces_sp._oral_taxon_414
Actinomyces_sp._oral_taxon_448
Actinomyces_timonensis
Atopobium_sp._oral_taxon_199
Campylobacter_concisus
Campylobacter_curvus
Campylobacter_rectus
Capnocytophaga_gingivalis
Capnocytophaga_granulosa
Capnocytophaga_haemolytica
Capnocytophaga_leadbetteri
Capnocytophaga_ochracea
Capnocytophaga_sp._oral_taxon_323
Capnocytophaga_sp._oral_taxon_324
Capnocytophaga_sp._oral_taxon_335
Capnocytophaga_sp._oral_taxon_380
Capnocytophaga_sp._oral_taxon_412
Capnocytophaga_sp._oral_taxon_878
Capnocytophaga_sputigena
Cryptobacterium_curtum
Cutibacterium_acnes
Dialister_pneumosintes
Fusobacterium_periodonticum
Haemophilus_pittmaniae
Kingella_oralis
Lachnoanaerobaculum_orale
Lachnoanaerobaculum_saburreum
Leptotrichia_sp._oral_taxon_215
Leptotrichia_sp._oral_taxon_417
Leptotrichia_wadei
Mogibacterium_diversum
Mogibacterium_neglectum
Mogibacterium_timidum
Neisseria_bacilliformis
Neisseria_perflava
Neisseria_polysaccharea
Oribacterium_asaccharolyticum
Oribacterium_sp._oral_taxon_078
Ottowia_sp._oral_taxon_894
Parvimonas_micra
Peptostreptococcaceae_bacterium_oral_taxon_113
Porphyromonas_sp._oral_taxon_275
Prevotella_jejuni
Prevotella_salivae
Prevotella_sp._oral_taxon_475
Prevotella_veroralis
Propionibacterium_acidifaciens
Pseudopropionibacterium_rubrum
Rothia_aeria
Scardovia_wiggsiae
Schaalia_meyeri
Selenomonas_sp._oral_taxon_133
Selenomonas_sp._oral_taxon_136
Selenomonas_sp._oral_taxon_478
Selenomonas_sputigena
Stomatobaculum_longum
Streptococcus_anginosus
Streptococcus_infantis
Streptococcus_peroris
Treponema_lecithinolyticum
Veillonella_sp._oral_taxon_780

Exclusively in progressing

Actinomyces_dentalis
Actinomyces_graevenitzii
Actinomyces_sp._oral_taxon_169
Actinomyces_sp._oral_taxon_171
Actinomyces_sp._oral_taxon_172
Aggregatibacter_segnis
Aggregatibacter_sp._oral_taxon_458
Alloprevotella_rava
Alloprevotella_tannerae
Atopobium_parvulum
Bacteroidetes_oral_taxon_274
Capnocytophaga_sp._oral_taxon_338
Capnocytophaga_sp._oral_taxon_864
Catonella_morbi
Eikenella_corrodens
Fusobacterium_naviforme
Fusobacterium_nucleatum
Gemella_morbillorum
Gemella_sanguinis
Leptotrichia_buccalis
Leptotrichia_sp._oral_taxon_498
Neisseria_cinerea
Neisseria_elongata
Neisseria_macacae
Neisseria_mucosa
Neisseria_oralis
Olsenella_sp._oral_taxon_807
Peptostreptococcus_stomatis
Prevotella_fusca
Prevotella_melaninogenica
Prevotella_oralis
Prevotella_shahii
Prevotella_sp._oral_taxon_317
Pseudopropionibacterium_propionicum
Schaalia_cardiffensis
Schaalia_georgiae
Selenomonas_artemidis
Streptococcus_cristatus
Streptococcus_lactarius
Streptococcus_parasanguinis
Streptococcus_sanguinis
Tannerella_sp._oral_taxon_HOT-286
TM7_phylum_sp._oral_taxon_350
TM7_phylum_sp._oral_taxon_352
TM7_phylum_sp._oral_taxon_356
TM7_phylum_sp._oral_taxon_957

Common elements in progressing and stable

Actinomyces_gerencseriae
Actinomyces_johnsonii
Actinomyces_massiliensis
Actinomyces_viscosus
Campylobacter_showae
Capnocytophaga_sp._oral_taxon_332
Corynebacterium_matruchotii
Fusobacterium_sp._oral_taxon_370
Granulicatella_elegans
Lachnoanaerobaculum_umeaense
Leptotrichia_hofstadii
Leptotrichia_hongkongensis
Leptotrichia_shahii
Leptotrichia_sp._oral_taxon_212
Leptotrichia_sp._oral_taxon_225
Neisseria_flavescens
Neisseria_subflava
Peptostreptococcus_anaerobius
Prevotella_maculosa
Prevotella_oris
Prevotella_oulorum
Prevotella_pleuritidis
Prevotella_saccharolytica
Prevotella_scopos
Rothia_dentocariosa
Schaalia_odontolytica
Selenomonas_noxia
Selenomonas_sp._oral_taxon_137
Selenomonas_sp._oral_taxon_149
Simonsiella_muelleri
Streptococcus_australis
Streptococcus_intermedius
Streptococcus_oralis
TM7_phylum_sp._oral_taxon_346
TM7_phylum_sp._oral_taxon_348
Veillonella_denticariosi
Veillonella_parvula
Veillonella_rogosae

Figure S2

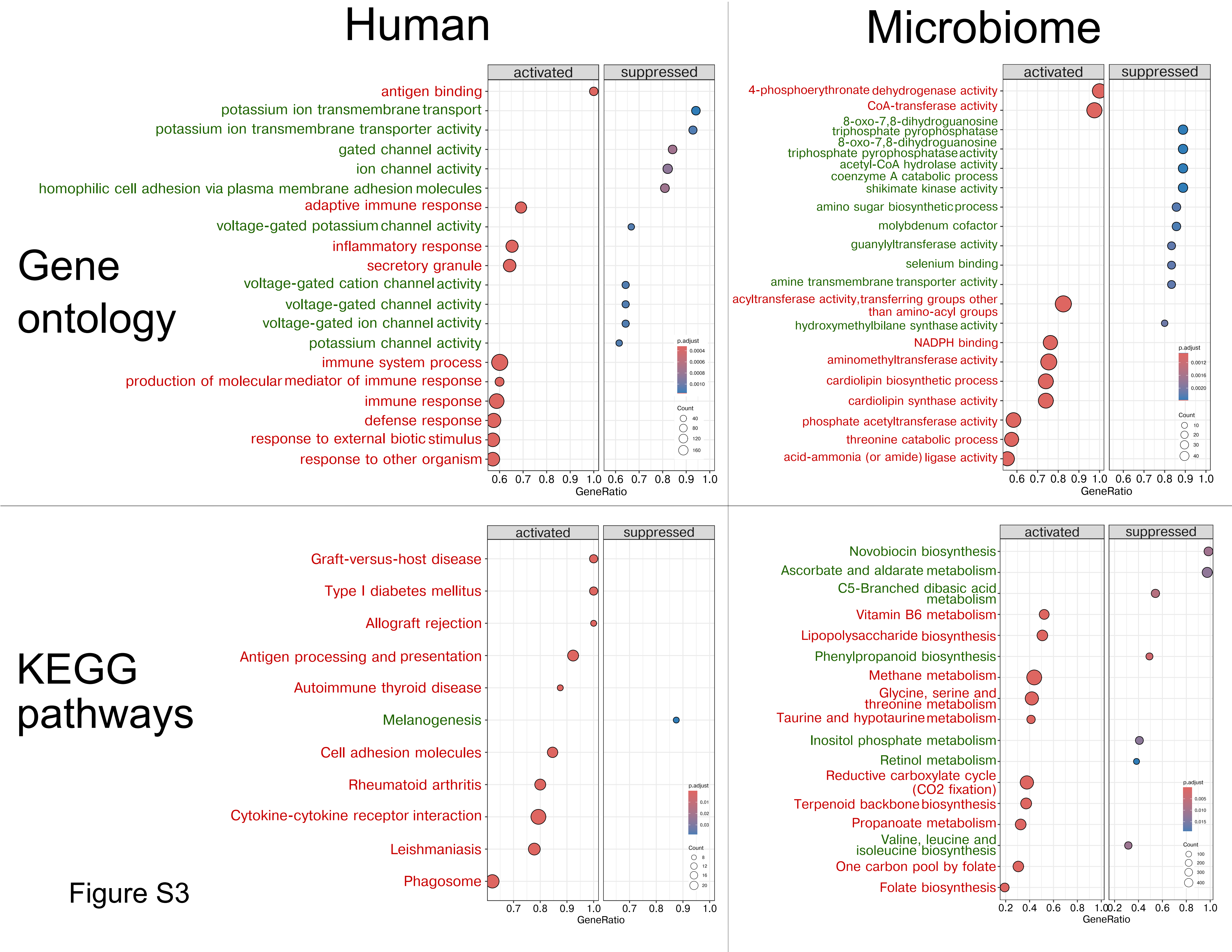

Human
Microbiome
Gene ontology
KEGG pathways
activated
suppressed
antigen binding
potassium ion transmembrane transport
potassium ion transmembrane transporter activity
gated channel activity
ion channel activity
homophilic cell adhesion via plasma membrane adhesion molecules
adaptive immune response
voltage-gated potassium channel activity
inflammatory response
secretory granule
voltage-gated cation channel activity
voltage-gated channel activity
voltage-gated ion channel activity
potassium channel activity
immune system process
production of molecular mediator of immune response
immune response
defense response
response to external biotic stimulus
response to other organism
p.adjust
Count
GeneRatio
4-phosphoerythronate dehydrogenase activity
CoA-transferase activity
8-oxo-7,8-dihydroguanosine triphosphate pyrophosphatase
8-oxo-7,8-dihydroguanosine triphosphate pyrophosphatase activity
acetyl-CoA hydrolase activity
coenzyme A catabolic process
shikimate kinase activity
amino sugar biosynthetic process
molybdenum cofactor
guanylyltransferase activity
selenium binding
amine transmembrane transporter activity
acyltransferase activity,transferring groups other than amino-acyl groups
hydroxymethylbilane synthase activity
NADPH binding
aminomethyltransferase activity
cardiolipin biosynthetic process
cardiolipin synthase activity
phosphate acetyltransferase activity
threonine catabolic process
acid-ammonia (or amide) ligase activity
Graft-versus-host disease
Type I diabetes mellitus
Allograft rejection
Antigen processing and presentation
Autoimmune thyroid disease
Melanogenesis
Cell adhesion molecules
Rheumatoid arthritis
Cytokine-cytokine receptor interaction
Leishmaniasis
Phagosome
Novobiocin biosynthesis
Ascorbate and aldarate metabolism
C5-Branched dibasic acid metabolism
Vitamin B6 metabolism
Lipopolysaccharide biosynthesis
Phenylpropanoid biosynthesis
Methane metabolism
Glycine, serine and threonine metabolism
Taurine and hypotaurine metabolism
Inositol phosphate metabolism
Retinol metabolism
Reductive carboxylate cycle (CO2 fixation)
Terpenoid backbone biosynthesis
Propanoate metabolism
Valine, leucine and isoleucine biosynthesis
One carbon pool by folate
Folate biosynthesis

Figure S3

Figure S4

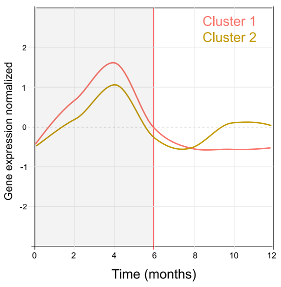

Metabolic and Biosynthetic Processes

General Metabolism:
- Aminoacyl-tRNA Editing Activity (3.04%)
- Ornithine Metabolic Process (2.17%)
- Tricarboxylic Acid Cycle (1.74%)
- Isopentenyl Diphosphate Biosynthetic Process (1.3%)
- Fatty Acid Metabolic Process (0.87%)
- Glycerol Metabolic Process (0.87%)
- Butanoate Metabolism (13.98%)
- Fatty Acid Metabolism (1.08%)
- Pyruvate Metabolism (1.08%)
- Methane Metabolism (1.08%)
- Amino Sugar and Nucleotide Sugar Metabolism (1.08%)
- Cysteine and Methionine Metabolism (1.08%)

Biosynthesis Processes:
- Lipopolysaccharide Biosynthetic Process (1.3%)
- CTP Biosynthetic Process (0.87%)
- Pyridoxal Phosphate Biosynthetic Process (0.87%)
- Heme O Biosynthetic Process (0.87%)
- Phosphoribosylanthranilate Isomerase Activity (0.87%)
- Glycosphingolipid Biosynthesis - Ganglio Series (2.15%)
- Streptomycin Biosynthesis (2.15%)
- Pantothenate and CoA Biosynthesis (1.08%)
- Sphingolipid Metabolism (1.08%)
- Fatty Acid Biosynthesis (1.08%)
- Ubiquinone and Other Terpenoid-Quinone Biosynthesis (1.08%)
- Lipopolysaccharide Biosynthesis (1.08%)
- Glycosylphosphatidylinositol (GPI)-Anchor Biosynthesis (1.08%)
- Glycerophospholipid Metabolism (1.08%)
- Lipid Biosynthetic Process (1.08%)

Catabolic Processes:
- Cellular Amino Acid Catabolic Process (0.87%)
- Peptidoglycan Catabolic Process (1.08%)

Transport and Binding Activities

Transport Activities:
- Cobalt Ion Transport (1.3%)
- Proton Transmembrane Transport (1.3%)
- Nicotinamide Mononucleotide Transmembrane Transporter Activity (0.87%)
- Symporter Activity (0.87%)
- Transmembrane Transporter Activity (0.87%)
- P-Type Potassium Transmembrane Transporter Activity (1.3%)
- Transporter Activity (2.15%)
- Nucleobase Transmembrane Transporter Activity (1.08%)
- Symporter Activity (1.08%)

Binding Activities:
- Cation Binding (0.87%)
- Manganese Ion Binding (0.87%)
- Flavin Adenine Dinucleotide Binding (1.08%)

Extrachromosomal DNA:
- Extrachromosomal Circular DNA (2.17%)

Enzymatic and Catalytic Activities

Enzyme-Specific Activities:
- 6-Carboxyhexanoate-CoA Ligase Activity (2.17%)
- UDP-Glucose 6-Dehydrogenase Activity (1.74%)
- Phenylalanyl-tRNA Aminoacylation (1.3%)
- Oxoglutarate Dehydrogenase (Succinyl-Transferring) Activity (1.3%)
- Guanylate Kinase Activity (1.3%)
- Glutamyl-tRNA Aminoacylation (1.3%)
- S-Ribosylhomocysteine Lyase Activity (0.87%)
- Glutaminase Activity (0.87%)
- Imidazoleglycerol-Phosphate Synthase Activity (0.87%)
- Carbon-Oxygen Lyase Activity (0.87%)
- Porphobilinogen Synthase Activity (0.87%)
- Pseudouridine Synthase Activity (2.15%)
- Excinuclease ABC Activity (2.15%)
- Acetyl-CoA Carboxylase Complex (2.15%)
- Phosphoribosylglycinamide Formyltransferase Activity (1.08%)
- Metalloendopeptidase Activity (1.08%)
- Aspartic-Type Endopeptidase Activity (1.08%)
- N-Methyltransferase Activity (1.08%)

ATP Synthase and Energy-Related Activities:
- Proton-Transporting ATP Synthase Complex, Coupling Factor F(o) (1.3%)
- NADH Dehydrogenase (Ubiquinone) Activity (0.87%)

Nucleic Acid and Genetic Processes

DNA and RNA-Related Activities:
- DNA Replication, Synthesis of RNA Primer (0.87%)
- Phosphorelay Sensor Kinase Activity (1.3%)
- Transcription, DNA-Templated (0.87%)
- DNA-Templated Transcription, Termination (0.87%)
- Regulation of DNA-Templated Transcription, Elongation (0.87%)
- Helicase Activity (0.87%)
- Translation Elongation Factor Activity (0.87%)
- Phosphorelay Sensor Kinase Activity (1.3%)
- DNA Integration (1.08%)
- DNA Binding (1.08%)
- DNA Modification (1.08%)
- DNA Recombination (1.08%)
- RNA Methylation (1.08%)
- rRNA Methylation (1.08%)
- Ribosome Biogenesis (1.08%)
- rRNA (Adenine-N6,N6-)-Dimethyltransferase Activity (1.08%)
- Maintenance of CRISPR Repeat Elements (1.08%)

Cellular and Structural Processes

Cellular Processes:
- Cell Wall Macromolecule Biosynthetic Process (0.87%)
- Proteasome Core Complex (0.87%)

Regulation of Signaling Pathways

Regulation and Signaling:
- Regulation of Vascular Associated Smooth Muscle Cell Migration (2.72%)
- Positive Regulation of Nitrogen Compound Metabolic Process (5.34%)
- Protein Modification Processes (5.44%)
- Phosphatidylinositol Signaling System (1.08%)
- Sulfur Metabolism (1.08%)
- Regulation of Transcription, DNA-Templated (1.08%)
- Phosphatidylinositol Signaling System (1.08%)
- Regulation of Transcription, DNA-Templated (1.08%)
- Regulation of DNA Repair (1.08%)
- Regulation of Cell Shape (1.08%)

Miscellaneous Processes

- Cysteine Export Across Plasma Membrane (1.3%)
- GTPase Activity (1.08%)
- Base-Excision Repair (1.08%)
- Transposase Activity (1.08%)
- Cell Wall Organization (1.08%)

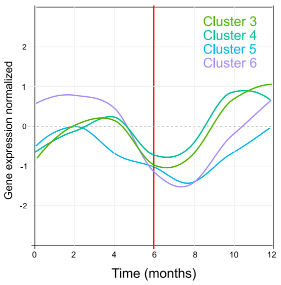

Metabolic and Biosynthetic Processes

General Metabolism:
- Butanoate Metabolism (13.98%)
- Fatty Acid Metabolism (1.08%)
- Pyruvate Metabolism (1.08%)
- Methane Metabolism (1.08%)
- Amino Sugar and Nucleotide Sugar Metabolism (1.08%)
- Cysteine and Methionine Metabolism (1.08%)

Biosynthesis Processes:
- Glycosphingolipid Biosynthesis - Ganglio Series (2.15%)
- Streptomycin Biosynthesis (2.15%)
- Pantothenate and CoA Biosynthesis (1.08%)
- Sphingolipid Metabolism (1.08%)
- Fatty Acid Biosynthesis (1.08%)
- Ubiquinone and Other Terpenoid-Quinone Biosynthesis (1.08%)
- Lipopolysaccharide Biosynthesis (1.08%)
- Glycosylphosphatidylinositol (GPI)-Anchor Biosynthesis (1.08%)
- Glycerophospholipid Metabolism (1.08%)
- Lipid Biosynthetic Process (1.08%)

Catabolic Processes:
- Peptidoglycan Catabolic Process (1.08%)

Enzymatic and Catalytic Activities

Enzyme-Specific Activities:
- Pseudouridine Synthase Activity (2.15%)
- Excinuclease ABC Activity (2.15%)
- Acetyl-CoA Carboxylase Complex (2.15%)
- Phosphoribosylglycinamide Formyltransferase Activity (1.08%)
- Metalloendopeptidase Activity (1.08%)
- Aspartic-Type Endopeptidase Activity (1.08%)
- N-Methyltransferase Activity (1.08%)

Nucleic Acid and Genetic Processes

DNA and RNA-Related Activities:
- DNA Integration (1.08%)
- DNA Binding (1.08%)
- DNA Modification (1.08%)
- DNA Recombination (1.08%)
- RNA Methylation (1.08%)
- rRNA Methylation (1.08%)
- Ribosome Biogenesis (1.08%)
- Maintenance of CRISPR Repeat Elements (1.08%)

Transport and Binding Activities

Transporter Activities:
- Transporter Activity (2.15%)
- Nucleobase Transmembrane Transporter Activity (1.08%)
- Symporter Activity (1.08%)

Binding Activities:
- Flavin Adenine Dinucleotide Binding (1.08%)

Regulation of Signaling Pathways

Regulation and Signaling:
- Phosphatidylinositol Signaling System (1.08%)
- Regulation of Transcription, DNA-Templated (1.08%)
- Regulation of DNA Repair (1.08%)
- Regulation of Cell Shape (1.08%)

Miscellaneous Processes

- GTPase Activity (1.08%)
- Base-Excision Repair (1.08%)
- Transposase Activity (1.08%)
- Cell Wall Organization (1.08%)

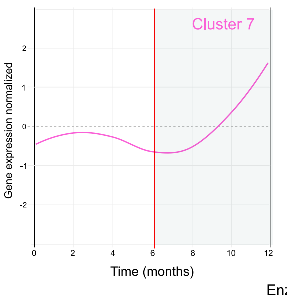

Metabolic and Biosynthetic Processes

Biosynthesis and Metabolism:
- Pteridine-Containing Compound Biosynthetic Process (3.33%)
- Pyrimidine Ribonucleotide Biosynthetic Process (3.33%)
- Spermidine Metabolic Process (2.22%)
- Carbohydrate Metabolic Process (2.22%)
- Small Molecule Biosynthetic Process (1.11%)
- Aminoacyl-tRNA Biosynthesis (1.11%)
- Pyridoxine Biosynthetic Process (1.11%)
- Cobalamin Biosynthetic Process (1.11%)
- Carbohydrate Biosynthetic Process (1.11%)
- Pyridine Nucleotide Biosynthetic Process (1.11%)
- 7-Methylguanosine Biosynthetic Process (1.11%)
- Tyrosine Metabolism (26.83%)
- Galactose Metabolism (4.88%)
- Fatty Acid Metabolism (2.44%)
- Beta-Alanine Metabolism (2.44%)
- Glycerolipid Metabolism (2.44%)
- Glutathione Metabolism (2.44%)
- Cysteine and Methionine Metabolism (2.44%)
- Fructose and Mannose Metabolism (2.44%)
- Starch and Sucrose Metabolism (2.44%)
- Amino Sugar and Nucleotide Sugar Metabolism (2.44%)
- Butanoate Metabolism (2.44%)
- Nucleobase-Containing Small Molecule Interconversion (2.44%)
- Pantothenate and CoA Biosynthesis (2.44%)
- Porphyrin-Containing Compound Biosynthetic Process (2.44%)
- Histidine Biosynthetic Process (2.44%)
- Lysine Biosynthesis (2.44%)

Enzymatic and Catalytic Activities

Enzyme-Specific Activities:
- N-Acyltransferase Activity (4.44%)
- Isoleucine-tRNA Ligase Activity (2.22%)
- Arginine-tRNA Ligase Activity (2.22%)
- 1,4-Dihydroxy-2-Naphthoate Octaprenyltransferase Activity (2.22%)
- Dihydroorotate Dehydrogenase Activity (2.22%)
- Carbamate Kinase Activity (1.11%)
- Quinolinate Synthetase A Activity (1.11%)
- tRNA (Guanine-N1-)-Methyltransferase Activity (1.11%)
- 6-Carboxy-5,6,7,8-Tetrahydropterin Synthase Activity (1.11%)
- Glycine C-Acetyltransferase Activity (1.11%)
- Protein-N(PI)-Phosphohistidine-Sugar Phosphotransferase Activity (2.44%)

Oxidoreductase and Related Activities:
- Aldehyde-Lyase Activity (1.11%)

Nucleic Acid and Genetic Processes

DNA and RNA-Related Activities:
- DNA Polymerase III Complex (3.33%)
- RNA Splicing (2.22%)
- DNA Topological Change (2.22%)
- Transcription, DNA-Templated (2.22%)
- mRNA Catabolic Process (1.11%)
- Selenocysteine Incorporation (1.11%)
- Base-Excision Repair (2.44%)

Response Mechanisms:
- SOS Response (2.22%)

Transport and Binding Activities

Transport and Ion Transport:
- ATPase-Coupled Transmembrane Transporter Activity (2.22%)
- Iron Ion Transport (1.11%)
- Xenobiotic Transmembrane Transporter Activity (1.11%)
- Nitrogen Compound Transport (1.11%)

Cellular Processes and Motility

Motility Activities:
- Cilium or Flagellum-Dependent Cell Motility (3.33%)
- Bacterial-Type Flagellum-Dependent Cell Motility (2.22%)
- Protein Targeting (2.22%)
- Chemotaxis (1.11%)

Structural Components:
- Bacterial-Type Flagellum Basal Body, MS Ring (1.11%)
- Protein-Containing Complex (1.11%)

Energy and Cellular Respiration

- ATP Hydrolysis Activity (1.11%)
- Protein-Transporting ATPase Activity (1.11%)
- ATP Binding (1.11%)
- Oxidative Phosphorylation (7.32%)
- NADH Dehydrogenase (Ubiquinone) Activity (2.44%)
- Citrate Cycle (TCA Cycle) (2.44%)
- Reductive Carboxylate Cycle (CO2 Fixation) (2.44%)
- Tricarboxylic Acid Cycle (2.44%)

Miscellaneous Processes

- Histidine Catabolic Process (1.11%)
- Electron Transport Chain (1.11%)
- Proteolysis (1.11%)
- Protein Demethylation (2.22%)
- Biotin Metabolism (2.44%)
- Riboflavin Metabolism (2.44%)
- Sulfur Metabolism (2.44%)

Figure S5

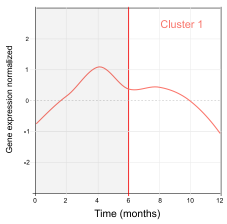

Metabolic and Biosynthetic Processes

General Metabolism:
- Fucose Catabolic Process (4.55%)
- Glyceraldehyde-3-Phosphate Metabolic Process (3.03%)
- Carbohydrate Metabolic Process (1.52%)
- Aspartate Family Amino Acid Biosynthetic Process (1.52%)
- Small Molecule Biosynthetic Process (1.52%)
- Lipid A Biosynthetic Process (1.52%)
- Carbohydrate Biosynthetic Process (1.52%)
- Asparagine Biosynthetic Process (1.52%)
- Steroid Biosynthesis (4.55%)
- Tyrosine Metabolism (27.27%)
- Oxidative Phosphorylation (13.64%)
- Sphingolipid Metabolism (4.55%)
- Glycerolipid Metabolism (4.55%)
- Pyruvate Metabolism (4.55%)
- Histidine Biosynthetic Process (4.55%)
- Arginine and Proline Metabolism (4.55%)
- Starch and Sucrose Metabolism (4.55%)

Enzymatic and Catalytic Activities

Enzyme-Specific Activities:
- Serine-Type Peptidase Activity (3.03%)
- Methylmalonyl-CoA Mutase Activity (3.03%)
- N-Acetylornithine Carbamoyltransferase Activity (1.52%)
- Ribonucleoside-Triphosphate Reductase Activity (1.52%)
- Corrinoid Adenosyltransferase Activity (3.03%)
- 3-Oxoacyl-[Acyl-Carrier-Protein] Synthase Activity (1.52%)
- Glycine C-Acetyltransferase Activity (3.03%)

Protein and Ribosomal Processes

Translation and Ribosome-Related Processes:
- Translation (6.06%)
- Translation Elongation Factor Activity (3.03%)
- Translation Release Factor Activity, Codon Specific (1.52%)
- Protein-Containing Complex Assembly (3.03%)
- Organellar Small Ribosomal Subunit (3.03%)
- Plastid Large Ribosomal Subunit (1.52%)
- Large Ribosomal Subunit (1.52%)
- 5S rRNA Binding (1.52%)

Transport and Binding Activities

Transport and Symporter Activities:
- Sodium Symporter Activity (3.03%)
- Cobalt Ion Transmembrane Transporter Activity (1.52%)

Sugar Transport and Processing:
- Phosphoenolpyruvate-Dependent Sugar Phosphotransferase System (4.55%)

Binding Activities:
- Protein Binding (1.52%)
- S-Adenosyl-L-Methionine Binding (1.52%)
- Carbohydrate Binding (1.52%)

Nucleic Acid and Genetic Processes

DNA and RNA-Related Activities:
- DNA-Templated Transcription, Termination (3.03%)
- mRNA Catabolic Process (1.52%)
- DNA Binding (4.55%)
- Regulation of Transcription, DNA-Templated (4.55%)
- Ribosome Biogenesis (4.55%)

Cellular and Structural Processes

Cellular Processes and Structures:
- Cell Outer Membrane (3.03%)
- FtsZ-Dependent Cytokinesis (1.52%)

Cellular and Motility Processes

Motility Activities:
- Bacterial-Type Flagellum-Dependent Cell Motility (1.52%)

Oxidative and Stress Responses

Oxidative Processes:
- Oxidoreductase Activity, Acting on the Aldehyde or Oxo Group of Donors (4.55%)
- Response to Oxidative Stress (1.52%)

Miscellaneous Processes

- Molecular Function (4.55%)
- Novobiocin Biosynthesis (4.55%)

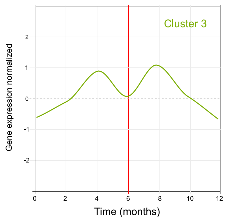

Metabolic and Biosynthetic Processes

General Metabolism:
- Tricarboxylic Acid Cycle (5.38%)
- Pyridoxine Biosynthetic Process (5.38%)
- Anaerobic Respiration (3.23%)
- Histidine Catabolic Process (2.15%)
- Dimethylallyl Diphosphate Biosynthetic Process (1.08%)
- Cobalamin Biosynthetic Process (1.08%)
- Glycogen Catabolic Process (1.08%)
- Lipoate Biosynthetic Process (3.23%)
- Cell Wall Macromolecule Biosynthetic Process (2.15%)
- Biosynthesis of Type II Polyketide Products (25.0%)
- Galactose Metabolism (8.33%)
- Glycerophospholipid Metabolism (8.33%)
- Pyruvate Metabolism (4.17%)
- Amino Sugar and Nucleotide Sugar Metabolism (4.17%)
- Fructose and Mannose Metabolism (4.17%)
- Nicotinate and Nicotinamide Metabolism (4.17%)
- Butanoate Metabolism (4.17%)
- Sulfur Metabolism (4.17%)
- Fatty Acid Metabolism (4.17%)
- Tryptophan Biosynthetic Process (4.17%)
- Pantothenate and CoA Biosynthesis (4.17%)

Protein and Ribosomal Processes

Ribosome and Protein Activities:
- Structural Constituent of Ribosome (2.15%)
- Ribosome (1.08%)
- Translation Release Factor Activity, Codon Specific (1.08%)
- Histidine-tRNA Ligase Activity (2.15%)
- Cysteine-tRNA Ligase Activity (2.15%)
- Phenylalanyl-tRNA Aminoacylation (3.23%)
- RNA Processing (3.23%)
- rRNA 2'-O-Methylation (2.15%)
- Polyribonucleotide Nucleotidyltransferase Activity (2.15%)
- Nascent Polypeptide-Associated Complex (2.15%)

Transport and Binding Activities

Binding Activities:
- NAD Binding (5.38%)
- Pyridoxal Phosphate Binding (1.08%)
- Copper Ion Binding (1.08%)
- ATP Binding (1.08%)
- Adenyl Nucleotide Binding (1.08%)

Transport Activities:
- Transmembrane Transport (1.08%)

Cellular and Motility Processes

Motility Activities:
- Cilium or Flagellum-Dependent Cell Motility (1.08%)
- Bacterial-Type Flagellum (1.08%)

Enzymatic and Catalytic Activities

Enzyme-Specific Activities:
- 4-Phosphoerythronate Dehydrogenase Activity (4.3%)
- Oxoglutarate Dehydrogenase (Succinyl-Transferring) Activity (3.23%)
- Carbon-Nitrogen Ligase Activity, with Glutamine as Amido-N-Donor (3.23%)
- Holo-[Acyl-Carrier-Protein] Synthase Activity (1.08%)
- 6-Carboxy-5,6,7,8-Tetrahydropterin Synthase Activity (1.08%)
- Methylmalonyl-CoA Mutase Activity (1.08%)
- 6-Phospho-Beta-Glucosidase Activity (1.08%)
- UDP-Glucose 6-Dehydrogenase Activity (1.08%)

Nucleic Acid and Genetic Processes

Regulation and DNA Processes:
- Regulation of DNA Replication (3.23%)
- Site-Specific Recombinase Activity (1.08%)
- Regulation of Transcription, DNA-Templated (4.17%)
- DNA Binding (4.17%)
- Maintenance of CRISPR Repeat Elements (4.17%)

Miscellaneous Processes

- Signal Transduction (1.08%)
- Glycine Decarboxylation via Glycine Cleavage System (1.08%)
- Nucleobase-Containing Compound Kinase Activity (2.15%)
- Oxidoreductase Activity, Acting on NAD(P)H, Quinone or Similar Compound as Acceptor (2.15%)
- Nucleoside Metabolic Process (4.17%)
- Transposase Activity (4.17%)

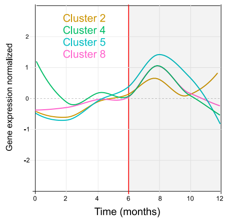

Metabolic and Biosynthetic Processes

General Metabolism:
- Methionine Catabolic Process (1.15%)
- Ornithine Metabolic Process (0.77%)
- L-Lysine Metabolic Process (0.77%)
- Fatty Acid Metabolic Process (0.77%)
- Glycerol Metabolic Process (0.77%)
- Nucleobase-Containing Compound Metabolic Process (0.77%)
- Proline Catabolic Process (0.77%)
- Butanoate Metabolism (10.62%)
- Fatty Acid Metabolism (2.5%)
- Fructose and Mannose Metabolism (0.62%)
- Glycerolipid Metabolism (0.62%)
- Amino Sugar and Nucleotide Sugar Metabolism (0.62%)
- Cysteine and Methionine Metabolism (0.62%)
- Arginine and Proline Metabolism (0.62%)
- Thiamine Metabolism (0.62%)
- Nicotinate and Nicotinamide Metabolism (0.62%)
- Glutathione Metabolism (0.62%)
- Lysine Degradation (0.62%)
- Purine Metabolism (0.62%)
- Starch and Sucrose Metabolism (0.62%)
- Inositol Phosphate Metabolism (0.62%)

Biosynthesis and Biosynthetic Pathways:
- Pyrimidine Ribonucleotide Biosynthetic Process (1.54%)
- Pyridoxine Biosynthetic Process (0.77%)
- Isopentenyl Diphosphate Biosynthetic Process, Methylerythritol 4-Phosphate Pathway (0.77%)
- CTP Biosynthetic Process (0.77%)
- Heme A Biosynthetic Process (0.77%)
- Pantothenate and CoA Biosynthesis (1.25%)
- Isoquinoline Alkaloid Biosynthesis (5.62%)
- Biosynthesis of Ansamycins (0.62%)
- Novobiocin Biosynthesis (0.62%)
- Lipopolysaccharide Biosynthesis (0.62%)
- Zeatin Biosynthesis (0.62%)
- Mo-Molybdopterin Cofactor Biosynthetic Process (0.62%)

Catabolic Processes:
- Cellular Amino Acid Catabolic Process (0.77%)
- Proteasomal Protein Catabolic Process (1.25%)
- Peptidoglycan Catabolic Process (0.62%)
- Branched-Chain Amino Acid Biosynthetic Process (0.62%)

Enzymatic and Catalytic Activities

Enzyme-Specific Activities:
- 4-Phosphoerythronate Dehydrogenase Activity (1.15%)
- Glutamate-tRNA Ligase Activity (1.15%)
- Quinolinate Synthetase A Activity (1.92%)
- Hydro-Lyase Activity (1.15%)
- Nitrate Reductase Complex (1.15%)
- Exonuclease ABC Activity (1.15%)
- Holo-[Acyl-Carrier-Protein] Synthase Activity (1.08%)
- Oxidoreductase Activity, Acting on the Aldehyde or Oxo Group of Donors, NAD or NADP as Acceptor (1.88%)
- Tryptophan Synthase Activity (1.25%)
- Pullulanase Activity (0.62%)
- Shikimate 3-Dehydrogenase (NADP+) Activity (0.62%)
- Peroxidase Activity (0.62%)
- Nucleoside Diphosphate Kinase Activity (0.62%)
- Cytidylate Kinase Activity (0.62%)

Nucleic Acid and Genetic Processes

DNA and RNA-Related Activities:
- DNA Topological Change (1.54%)
- DNA Polymerase III Complex (1.15%)
- Regulation of DNA-Templated Transcription, Elongation (0.77%)
- DNA Repair (0.77%)
- DNA Binding (0.62%)
- DNA Strand Exchange Activity (0.62%)
- DNA Integration (0.62%)
- DNA Methylation (0.62%)
- Base-Excision Repair (0.62%)
- Double-Strand Break Repair via Homologous Recombination (0.62%)
- Regulation of DNA Repair (0.62%)
- DNA Modification (0.62%)
- RNA Methylation (0.62%)

RNA Processing and Splicing:
- RNA Modification (0.77%)
- RNA Splicing (0.77%)
- Translation Initiation Factor Activity (0.77%)
- Translation Release Factor Activity (0.77%)
- Translational Elongation (0.77%)

Transport and Binding Activities

Transport Activities:
- P-Type Potassium Transmembrane Transporter Activity (1.15%)
- Sodium Ion Transport (0.77%)
- Xenobiotic Transmembrane Transport (1.15%)
- Nicotinate and Nicotinamide Transporter Activity (0.62%)
- Sodium Ion Transport (0.77%)

Binding Activities:
- Nucleotide Binding (2.69%)
- 4Fe-4S Binding (0.77%)
- NAD Binding (0.62%)
- FMN Binding (0.62%)

Cellular and Structural Processes

Cellular Processes:
- Bacterial-Type Flagellum-Dependent Cell Motility (1.54%)
- Protein Targeting (0.77%)
- Stringent Response (0.77%)
- Cytokinesis (0.62%)

Structural Components:
- Structural Constituent of Ribosome (1.54%)
- Cell Wall Organization (1.25%)

Miscellaneous Processes

- ATPase-Coupled Transmembrane Transporter Activity (0.77%)
- Transketolase Activity (0.77%)
- 4-(Cytidine 5'-Diphospho)-2-C-Methyl-D-Erythritol Kinase Activity (0.77%)
- Protein Dephosphorylation (0.62%)
- Transposase Activity (0.62%)
- Maintenance of CRISPR Repeat Elements (0.62%)

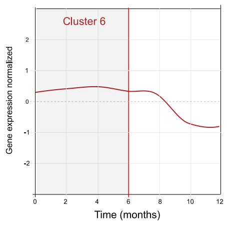

Protein and Ribosomal Processes

Ribosome Structure and Activity:
- Structural Constituent of Ribosome (8.0%)
- Cytosolic Small Ribosomal Subunit (4.0%)
- Plastid Large Ribosomal Subunit (2.0%)
- Ribosome (2.0%)

Translation and RNA Processes:
- Translation Elongation Factor Activity (4.0%)
- Translation Initiation Factor Activity (4.0%)
- rRNA Binding (4.0%)
- DNA-Directed 5'-3' RNA Polymerase Activity (4.0%)

Protein Modification and Transport:
- Protein Lipidation (6.0%)
- Post-Translational Protein Modification (2.0%)
- Protein Insertion Into Membrane (2.0%)
- Protein Binding (2.0%)

Peptidase and Enzymatic Activities:
- Serine-Type D-Ala-D-Ala Carboxypeptidase Activity (4.0%)
- Proteolysis (4.0%)
- Peptidase Activity (2.0%)
- Endoribonuclease Activity (2.0%)
- Phosphotransferase Activity, For Other Substituted Phosphate Groups (2.0%)

Metabolic and Biosynthetic Processes

Metabolic Pathways:
- Tetrahydrofolate Interconversion (2.0%)
- Rhamnose Metabolic Process (4.0%)
- Glycine Catabolic Process (2.0%)
- Menaquinone Biosynthetic Process (2.0%)
- AMP Salvage (4.0%)
- Cyclic Nucleotide Biosynthetic Process (4.0%)

Nitrogen and Carbon Metabolism:
- Nitrogen Metabolism (11.11%)
- Reductive Carboxylate Cycle (CO2 Fixation) (11.11%)
- Citrate Cycle (TCA Cycle) (11.11%)

Sugar and Carbohydrate Metabolism:
- Amino Sugar and Nucleotide Sugar Metabolism (11.11%)
- Galactose Metabolism (11.11%)

Transport and Binding Activities

Transport Activities:
- Organic Substance Transport (2.0%)
- Inorganic Cation Transmembrane Transport (2.0%)
- Efflux Transmembrane Transporter Activity (2.0%)

Electron Transfer and Chain:
- Electron Transfer Activity (2.0%)
- Electron Transport Chain (2.0%)

Phosphotransferase Systems:
- Phosphoenolpyruvate-Dependent Sugar Phosphotransferase System (11.11%)

Cellular and Motility Processes

Cellular Components:
- Cell Outer Membrane (2.0%)
- Extracellular Region (2.0%)

Motility and Channels:
- Bacterial-Type Flagellum-Dependent Cell Motility (2.0%)
- Channel Activity (2.0%)

Energy and Phosphorylation Processes

Energy Generation:
- Oxidative Phosphorylation (11.11%)
- Proton-Transporting ATP Synthase Activity, Rotational Mechanism (11.11%)

Nucleic Acid and Genetic Processes

Transcriptional Regulation:
- Regulation of Transcription, DNA-Templated (11.11%)

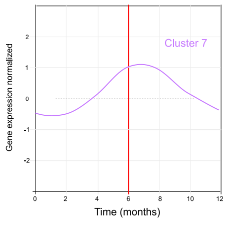

Metabolic and Biosynthetic Processes

General Metabolism:
- Extrachromosomal Circular DNA (4.65%)
- Methionine Catabolic Process (2.33%)
- D-Amino Acid Metabolism (1.55%)
- L-Lysine Metabolic Process (1.55%)
- Lipoate Biosynthetic Process (1.55%)
- Queuosine Biosynthetic Process (1.55%)
- Heme O Biosynthetic Process (1.55%)
- Lipopolysaccharide Biosynthesis (15.71%)
- Fatty Acid Metabolism (4.29%)
- Methane Metabolism (4.29%)
- Sulfur Metabolism (2.86%)
- Biotin Metabolism (2.86%)
- Beta-Alanine Metabolism (1.43%)
- Cysteine and Methionine Metabolism (1.43%)
- Pantothenate and CoA Biosynthesis (1.43%)
- Lysine Biosynthesis (1.43%)
- Thiamine Metabolism (1.43%)
- Amino Sugar and Nucleotide Sugar Metabolism (1.43%)
- Glycerolipid Metabolism (1.43%)
- Glycerophospholipid Metabolism (1.43%)
- Sphingolipid Metabolism (1.43%)
- Butanoate Metabolism (1.43%)
- Flavonoid Biosynthesis (5.71%)
- Terpenoid Backbone Biosynthesis (1.43%)
- Various Types of N-Glycan Biosynthesis (1.43%)
- Porphyrin-Containing Compound Biosynthetic Process (1.43%)
- Biosynthesis of Ansamycins (1.43%)
- Streptomycin Biosynthesis (1.43%)

Carbohydrate Metabolism:
- Galactose Metabolism (1.43%)
- Fructose and Mannose Metabolism (1.43%)
- Starch and Sucrose Metabolism (1.43%)
- Pentose-Phosphate Shunt (1.43%)
- Pentose Phosphate Pathway (1.43%)
- Glycolysis / Gluconeogenesis (1.43%)

Cycle and Pathway Processes:
- Citrate Cycle (TCA Cycle) (1.43%)
- Tricarboxylic Acid Cycle (1.43%)

Biosynthetic and Enzyme Activities:
- UDP-Glucose 6-Dehydrogenase Activity (3.88%)
- UDP-N-Acetyl-D-Mannosamine Dehydrogenase Activity (1.55%)

Protein and Ribosomal Processes

Ribosomal Activities:
- Structural Constituent of Ribosome (3.88%)
- Cytosolic Small Ribosomal Subunit (2.33%)
- Small Ribosomal Subunit (1.55%)
- Translational Elongation (1.55%)

Protein and Peptide Processes:
- Signal Peptide Processing (3.1%)
- Aminopeptidase Activity (1.55%)

Transport and Binding Activities

Transport Activities:
- Cobalt Ion Transmembrane Transporter Activity (2.33%)
- Ferrous Iron Transmembrane Transporter Activity (1.55%)
- Potassium Ion Transmembrane Transporter Activity (1.55%)

Transport Systems:
- ATP-Binding Cassette (ABC) Transporter Complex (2.86%)
- Protein-N(PI)-Phosphohistidine-Sugar Phosphotransferase System (1.43%)

Binding Activities:
- Ribonucleoside Binding (2.33%)
- NADP Binding (1.43%)

Nucleic Acid and Genetic Processes

DNA and RNA-Related Activities:
- DNA-Templated Transcription, Termination (2.33%)
- mRNA Catabolic Process (1.55%)
- DNA Modification (1.43%)
- Base-Excision Repair (1.43%)
- Exonuclease ABC Activity (1.43%)
- Maintenance of CRISPR Repeat Elements (1.43%)
- DNA-Templated Transcription Regulation (1.43%)

RNA Processing and Synthesis:
- tRNA Processing (2.86%)
- Pseudouridine Synthase Activity (1.43%)

Enzymatic and Catalytic Activities

Enzyme-Specific Activities:
- Exodeoxyribonuclease VII Complex (2.33%)
- Transposase Activity (1.43%)
- Methyltransferase Activity (1.43%)
- Phosphoenolpyruvate-Dependent Sugar Phosphotransferase System (1.43%)
- Exonuclease and Endonuclease Activities (1.43%)

Cellular and Motility Processes

Motility Activities:
- Cilium or Flagellum-Dependent Cell Motility (1.55%)

Miscellaneous Processes

- Electron Transport Chain (2.33%)
- Histidine Biosynthetic Process (1.43%)
- Tryptophan Biosynthetic Process (1.43%)
- Nucleobase-Containing Small Molecule Interconversion (1.43%)
- Phosphatidylinositol Signaling System (1.43%)

Figure S6

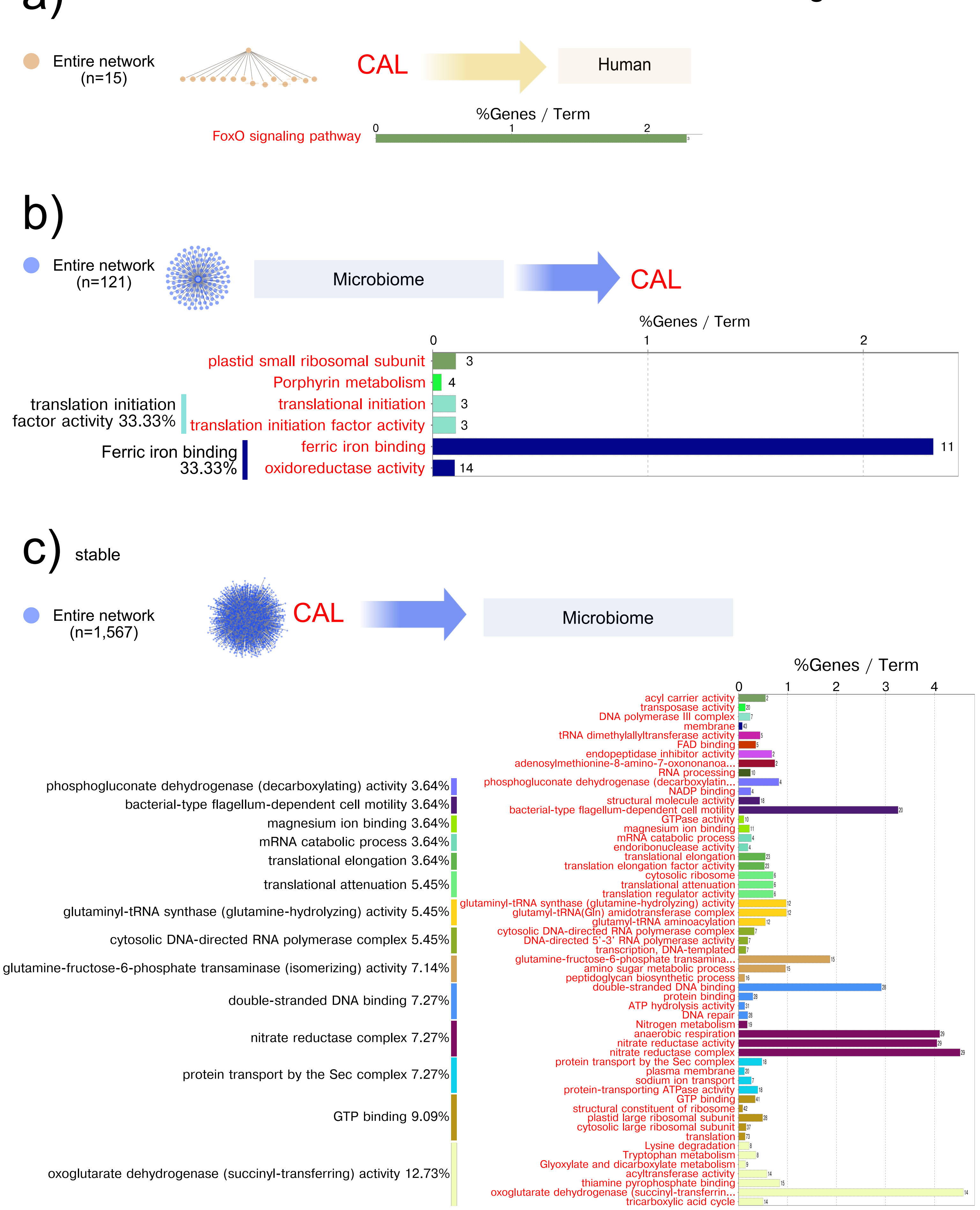
a)
stable
Entire network
(n=15)
CAL
Human
%Genes / Term
0
1
2
FoxO signaling pathway
b)
Entire network
(n=121)
Microbiome
CAL
%Genes / Term
0
1
2
plastid small ribosomal subunit
3
Porphyrin metabolism
4
translational initiation
3
translation initiation factor activity
3
ferric iron binding
11
oxidoreductase activity
14
translation initiation factor activity 33.33%
Ferric iron binding 33.33%
c)
stable
Entire network
(n=1,567)
CAL
Microbiome
%Genes / Term
0
1
2
3
4
acyl carrier activity
transposase activity
DNA polymerase III complex
membrane
tRNA dimethylallyltransferase activity
FAD binding
endopeptidase inhibitor activity
adenosylmethionine-8-amino-7-oxononanoa...
RNA processing
phosphogluconate dehydrogenase (decarboxylatin...
NADP binding
structural molecule activity
bacterial-type flagellum-dependent cell motility
GTPase activity
magnesium ion binding
mRNA catabolic process
endoribonuclease activity
translational elongation
translation elongation factor activity
cytosolic ribosome
translational attenuation
translation regulator activity
glutaminyl-tRNA synthase (glutamine-hydrolyzing) activity
glutamyl-tRNA(Gln) amidotransferase complex
glutamyl-tRNA aminoacylation
cytosolic DNA-directed RNA polymerase complex
DNA-directed 5'-3' RNA polymerase activity
transcription, DNA-templated
glutamine-fructose-6-phosphate transamina...
amino sugar metabolic process
peptidoglycan biosynthetic process
double-stranded DNA binding
protein binding
ATP hydrolysis activity
DNA repair
Nitrogen metabolism
anaerobic respiration
nitrate reductase activity
nitrate reductase complex
protein transport by the Sec complex
plasma membrane
sodium ion transport
protein-transporting ATPase activity
GTP binding
structural constituent of ribosome
plastid large ribosomal subunit
cytosolic large ribosomal subunit
translation
Lysine degradation
Tryptophan metabolism
Glyoxylate and dicarboxylate metabolism
acyltransferase activity
thiamine pyrophosphate binding
oxoglutarate dehydrogenase (succinyl-transferrin...
tricarboxylic acid cycle
phosphogluconate dehydrogenase (decarboxylating) activity 3.64%
bacterial-type flagellum-dependent cell motility 3.64%
magnesium ion binding 3.64%
mRNA catabolic process 3.64%
translational elongation 3.64%
translational attenuation 5.45%
glutaminyl-tRNA synthase (glutamine-hydrolyzing) activity 5.45%
cytosolic DNA-directed RNA polymerase complex 5.45%
glutamine-fructose-6-phosphate transaminase (isomerizing) activity 7.14%
double-stranded DNA binding 7.27%
nitrate reductase complex 7.27%
protein transport by the Sec complex 7.27%
GTP binding 9.09%
oxoglutarate dehydrogenase (succinyl-transferring) activity 12.73%

a)

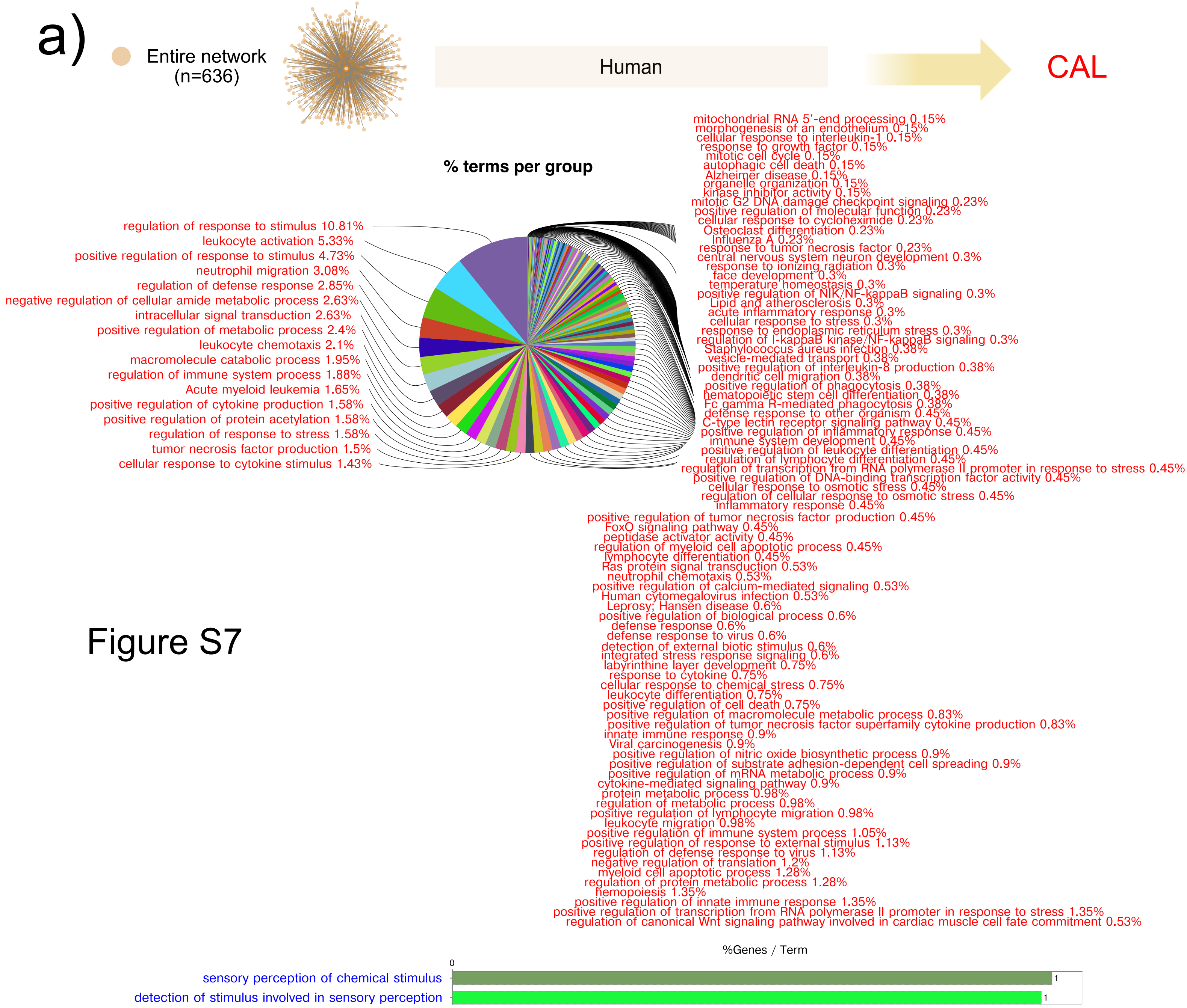

Entire network
(n=636)
Human
CAL
% terms per group
regulation of response to stimulus 10.81%
leukocyte activation 5.33%
positive regulation of response to stimulus 4.73%
neutrophil migration 3.08%
regulation of defense response 2.85%
negative regulation of cellular amide metabolic process 2.63%
intracellular signal transduction 2.63%
positive regulation of metabolic process 2.4%
leukocyte chemotaxis 2.1%
macromolecule catabolic process 1.95%
regulation of immune system process 1.88%
Acute myeloid leukemia 1.65%
positive regulation of cytokine production 1.58%
positive regulation of protein acetylation 1.58%
regulation of response to stress 1.58%
tumor necrosis factor production 1.5%
cellular response to cytokine stimulus 1.43%
mitochondrial RNA 5'-end processing 0.15%
morphogenesis of an endothelium 0.15%
cellular response to interleukin-1 0.15%
response to growth factor 0.15%
mitotic cell cycle 0.15%
autophagic cell death 0.15%
Alzheimer disease 0.15%
organelle organization 0.15%
kinase inhibitor activity 0.15%
mitotic G2 DNA damage checkpoint signaling 0.23%
positive regulation of molecular function 0.23%
cellular response to cycloheximide 0.23%
Osteoclast differentiation 0.23%
Influenza A 0.23%
response to tumor necrosis factor 0.23%
central nervous system neuron development 0.3%
response to ionizing radiation 0.3%
face development 0.3%
temperature homeostasis 0.3%
positive regulation of NIK/NF-kappaB signaling 0.3%
Lipid and atherosclerosis 0.3%
acute inflammatory response 0.3%
cellular response to stress 0.3%
response to endoplasmic reticulum stress 0.3%
regulation of I-kappaB kinase/NF-kappaB signaling 0.3%
Staphylococcus aureus infection 0.38%
vesicle-mediated transport 0.38%
positive regulation of interleukin-8 production 0.38%
dendritic cell migration 0.38%
positive regulation of phagocytosis 0.38%
hematopoietic stem cell differentiation 0.38%
Fc gamma R-mediated phagocytosis 0.38%
defense response to other organism 0.45%
C-type lectin receptor signaling pathway 0.45%
positive regulation of inflammatory response 0.45%
immune system development 0.45%
positive regulation of leukocyte differentiation 0.45%
regulation of lymphocyte differentiation 0.45%
regulation of transcription from RNA polymerase II promoter in response to stress 0.45%
positive regulation of DNA-binding transcription factor activity 0.45%
cellular response to osmotic stress 0.45%
regulation of cellular response to osmotic stress 0.45%
inflammatory response 0.45%
positive regulation of tumor necrosis factor production 0.45%
FoxO signaling pathway 0.45%
peptidase activator activity 0.45%
regulation of myeloid cell apoptotic process 0.45%
lymphocyte differentiation 0.45%
Ras protein signal transduction 0.53%
neutrophil chemotaxis 0.53%
positive regulation of calcium-mediated signaling 0.53%
Human cytomegalovirus infection 0.53%
Leprosy; Hansen disease 0.6%
positive regulation of biological process 0.6%
defense response 0.6%
defense response to virus 0.6%
detection of external biotic stimulus 0.6%
integrated stress response signaling 0.6%
labyrinthine layer development 0.75%
response to cytokine 0.75%
cellular response to chemical stress 0.75%
leukocyte differentiation 0.75%
positive regulation of cell death 0.75%
positive regulation of macromolecule metabolic process 0.83%
positive regulation of tumor necrosis factor superfamily cytokine production 0.83%
innate immune response 0.9%
Viral carcinogenesis 0.9%
positive regulation of nitric oxide biosynthetic process 0.9%
positive regulation of substrate adhesion-dependent cell spreading 0.9%
positive regulation of mRNA metabolic process 0.9%
cytokine-mediated signaling pathway 0.9%
protein metabolic process 0.98%
regulation of metabolic process 0.98%
positive regulation of lymphocyte migration 0.98%
leukocyte migration 0.98%
positive regulation of immune system process 1.05%
positive regulation of response to external stimulus 1.13%
regulation of defense response to virus 1.13%
negative regulation of translation 1.2%
myeloid cell apoptotic process 1.28%
regulation of protein metabolic process 1.28%
hemopoiesis 1.35%
positive regulation of innate immune response 1.35%
positive regulation of transcription from RNA polymerase II promoter in response to stress 1.35%
regulation of canonical Wnt signaling pathway involved in cardiac muscle cell fate commitment 0.53%
%Genes / Term
0
sensory perception of chemical stimulus
1
detection of stimulus involved in sensory perception
1

Figure S7

b)

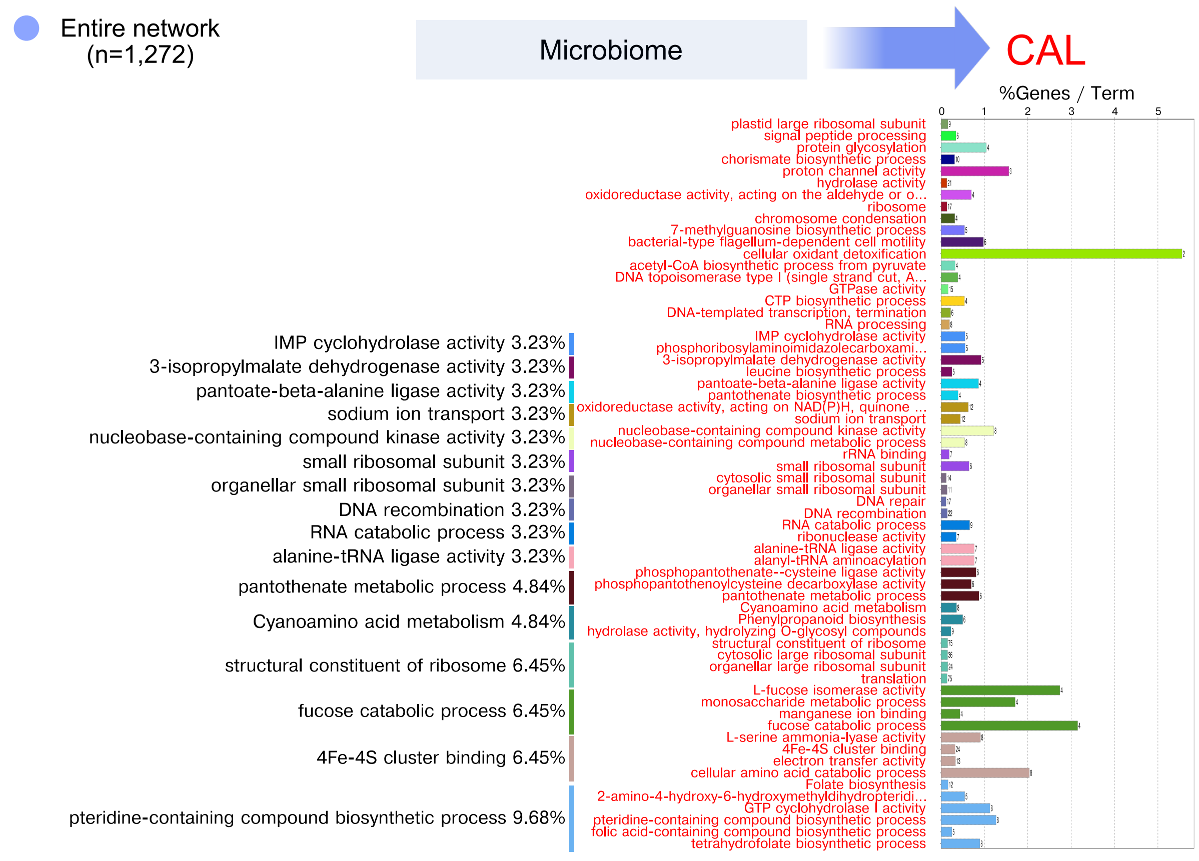

Entire network
(n=1,272)
Microbiome
CAL
%Genes / Term
0
1
2
3
4
5
IMP cyclohydrolase activity 3.23%
3-isopropylmalate dehydrogenase activity 3.23%
pantoate-beta-alanine ligase activity 3.23%
sodium ion transport 3.23%
nucleobase-containing compound kinase activity 3.23%
small ribosomal subunit 3.23%
organellar small ribosomal subunit 3.23%
DNA recombination 3.23%
RNA catabolic process 3.23%
alanine-tRNA ligase activity 3.23%
pantothenate metabolic process 4.84%
Cyanoamino acid metabolism 4.84%
structural constituent of ribosome 6.45%
fucose catabolic process 6.45%
4Fe-4S cluster binding 6.45%
pteridine-containing compound biosynthetic process 9.68%
plastid large ribosomal subunit
signal peptide processing
protein glycosylation
chorismate biosynthetic process
proton channel activity
hydrolase activity
oxidoreductase activity, acting on the aldehyde or o...
ribosome
chromosome condensation
7-methylguanosine biosynthetic process
bacterial-type flagellum-dependent cell motility
cellular oxidant detoxification
acetyl-CoA biosynthetic process from pyruvate
DNA topoisomerase type I (single strand cut, A...
GTPase activity
CTP biosynthetic process
DNA-templated transcription, termination
RNA processing
IMP cyclohydrolase activity
phosphoribosylaminoimidazolecarboxami...
3-isopropylmalate dehydrogenase activity
leucine biosynthetic process
pantoate-beta-alanine ligase activity
pantothenate biosynthetic process
oxidoreductase activity, acting on NAD(P)H, quinone ...
sodium ion transport
nucleobase-containing compound kinase activity
nucleobase-containing compound metabolic process
rRNA binding
small ribosomal subunit
cytosolic small ribosomal subunit
organellar small ribosomal subunit
DNA repair
DNA recombination
RNA catabolic process
ribonuclease activity
alanine-tRNA ligase activity
alanyl-tRNA aminoacylation
phosphopantothenate--cysteine ligase activity
phosphopantothenoylcysteine decarboxylase activity
pantothenate metabolic process
Cyanoamino acid metabolism
Phenylpropanoid biosynthesis
hydrolase activity, hydrolyzing O-glycosyl compounds
structural constituent of ribosome
cytosolic large ribosomal subunit
organellar large ribosomal subunit
translation
L-fucose isomerase activity
monosaccharide metabolic process
manganese ion binding
fucose catabolic process
L-serine ammonia-lyase activity
4Fe-4S cluster binding
electron transfer activity
cellular amino acid catabolic process
Folate biosynthesis
2-amino-4-hydroxy-6-hydroxymethyldihydropteridi...
GTP cyclohydrolase I activity
pteridine-containing compound biosynthetic process
folic acid-containing compound biosynthetic process
tetrahydrofolate biosynthetic process

Figure S8

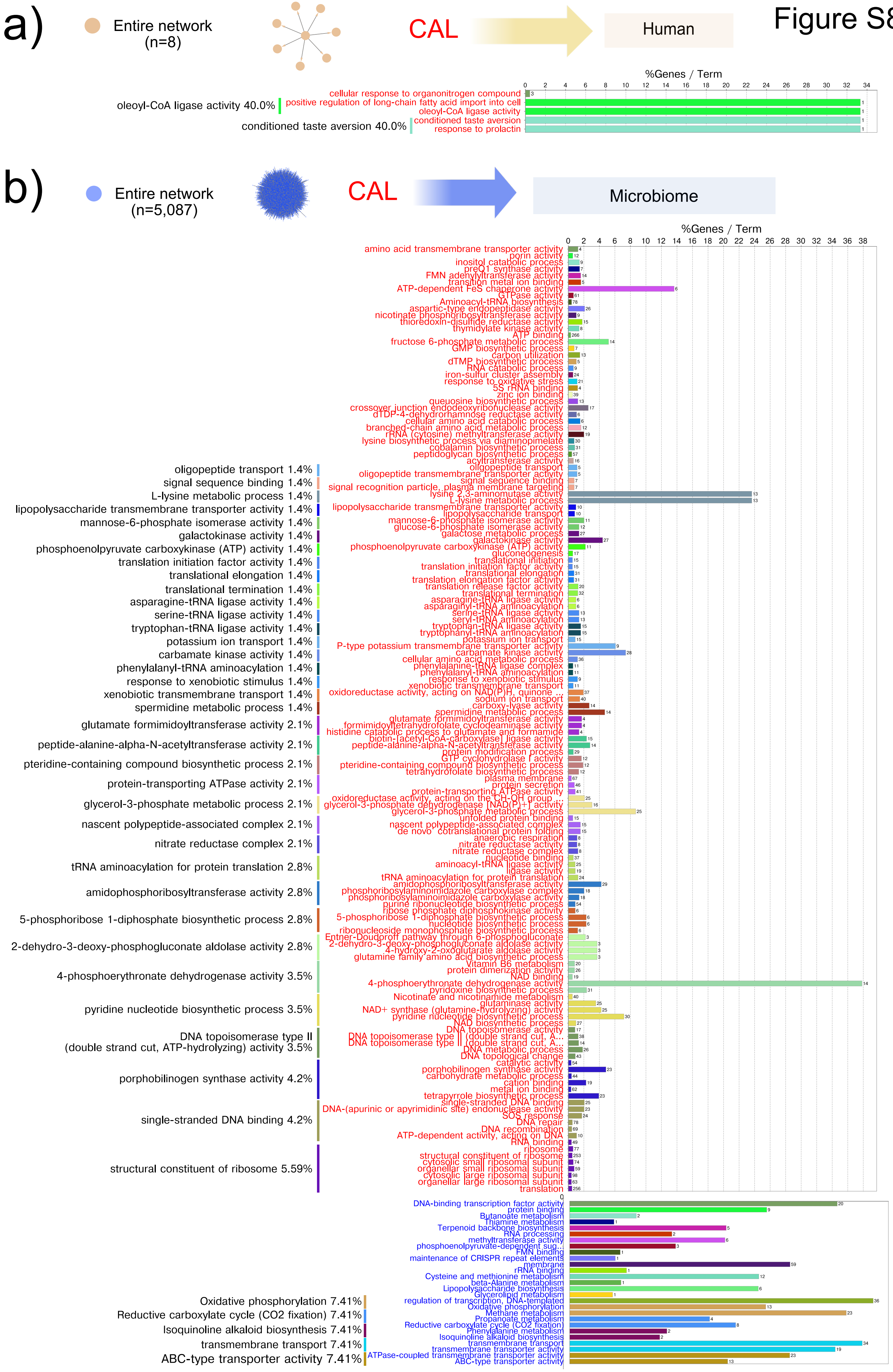

a)
Entire network
(n=8)
CAL
Human
%Genes / Term
oleoyl-CoA ligase activity 40.0%
conditioned taste aversion 40.0%
cellular response to organonitrogen compound
positive regulation of long-chain fatty acid import into cell
oleoyl-CoA ligase activity
conditioned taste aversion
response to prolactin
b)
Entire network
(n=5,087)
CAL
Microbiome
%Genes / Term
amino acid transmembrane transporter activity
porin activity
inositol catabolic process
preQ1 synthase activity
FMN adenylyltransferase activity
transition metal ion binding
ATP-dependent FeS chaperone activity
GTPase activity
Aminoacyl-tRNA biosynthesis
aspartic-type endopeptidase activity
nicotinate phosphoribosyltransferase activity
thioredoxin-disulfide reductase activity
thymidylate kinase activity
ATP binding
fructose 6-phosphate metabolic process
GMP biosynthetic process
carbon utilization
dTMP biosynthetic process
RNA catabolic process
iron-sulfur cluster assembly
response to oxidative stress
5S rRNA binding
zinc ion binding
queuosine biosynthetic process
crossover junction endodeoxyribonuclease activity
dTDP-4-dehydrorhamnose reductase activity
cellular amino acid catabolic process
branched-chain amino acid metabolic process
tRNA (cytosine) methyltransferase activity
lysine biosynthetic process via diaminopimelate
cobalamin biosynthetic process
peptidoglycan biosynthetic process
acyltransferase activity
oligopeptide transport 1.4%
signal sequence binding 1.4%
L-lysine metabolic process 1.4%
lipopolysaccharide transmembrane transporter activity 1.4%
mannose-6-phosphate isomerase activity 1.4%
galactokinase activity 1.4%
phosphoenolpyruvate carboxykinase (ATP) activity 1.4%
translation initiation factor activity 1.4%
translational elongation 1.4%
translational termination 1.4%
asparagine-tRNA ligase activity 1.4%
serine-tRNA ligase activity 1.4%
tryptophan-tRNA ligase activity 1.4%
potassium ion transport 1.4%
carbamate kinase activity 1.4%
phenylalanyl-tRNA aminoacylation 1.4%
response to xenobiotic stimulus 1.4%
xenobiotic transmembrane transport 1.4%
spermidine metabolic process 1.4%
glutamate formimidoyltransferase activity 2.1%
peptide-alanine-alpha-N-acetyltransferase activity 2.1%
pteridine-containing compound biosynthetic process 2.1%
protein-transporting ATPase activity 2.1%
glycerol-3-phosphate metabolic process 2.1%
nascent polypeptide-associated complex 2.1%
nitrate reductase complex 2.1%
tRNA aminoacylation for protein translation 2.8%
amidophosphoribosyltransferase activity 2.8%
5-phosphoribose 1-diphosphate biosynthetic process 2.8%
2-dehydro-3-deoxy-phosphogluconate aldolase activity 2.8%
4-phosphoerythronate dehydrogenase activity 3.5%
pyridine nucleotide biosynthetic process 3.5%
DNA topoisomerase type II
(double strand cut, ATP-hydrolyzing) activity 3.5%
porphobilinogen synthase activity 4.2%
single-stranded DNA binding 4.2%
structural constituent of ribosome 5.59%
oligopeptide transport
oligopeptide transmembrane transporter activity
signal sequence binding
signal recognition particle, plasma membrane targeting
lysine 2,3-aminomutase activity
L-lysine metabolic process
lipopolysaccharide transmembrane transporter activity
lipopolysaccharide transport
mannose-6-phosphate isomerase activity
glucose-6-phosphate isomerase activity
galactose metabolic process
galactokinase activity
phosphoenolpyruvate carboxykinase (ATP) activity
gluconeogenesis
translational initiation
translation initiation factor activity
translational elongation
translation elongation factor activity
translation release factor activity
translational termination
asparagine-tRNA ligase activity
asparaginyl-tRNA aminoacylation
serine-tRNA ligase activity
seryl-tRNA aminoacylation
tryptophan-tRNA ligase activity
tryptophanyl-tRNA aminoacylation
potassium ion transport
P-type potassium transmembrane transporter activity
carbamate kinase activity
cellular amino acid metabolic process
phenylalanine-tRNA ligase complex
phenylalanyl-tRNA aminoacylation
response to xenobiotic stimulus
xenobiotic transmembrane transport
oxidoreductase activity, acting on NAD(P)H, quinone ...
sodium ion transport
carboxy-lyase activity
spermidine metabolic process
glutamate formimidoyltransferase activity
formimidoyltetrahydrofolate cyclodeaminase activity
histidine catabolic process to glutamate and formamide
biotin-[acetyl-CoA-carboxylase] ligase activity
peptide-alanine-alpha-N-acetyltransferase activity
protein modification process
GTP cyclohydrolase I activity
pteridine-containing compound biosynthetic process
tetrahydrofolate biosynthetic process
plasma membrane
protein secretion
protein-transporting ATPase activity
oxidoreductase activity, acting on the CH-OH group ...
glycerol-3-phosphate dehydrogenase [NAD(P)+] activity
glycerol-3-phosphate metabolic process
unfolded protein binding
nascent polypeptide-associated complex
'de novo' cotranslational protein folding
anaerobic respiration
nitrate reductase activity
nitrate reductase complex
nucleotide binding
aminoacyl-tRNA ligase activity
ligase activity
tRNA aminoacylation for protein translation
amidophosphoribosyltransferase activity
phosphoribosylaminoimidazole carboxylase complex
phosphoribosylaminoimidazole carboxylase activity
purine ribonucleotide biosynthetic process
ribose phosphate diphosphokinase activity
5-phosphoribose 1-diphosphate biosynthetic process
nucleotide biosynthetic process
ribonucleoside monophosphate biosynthetic process
Entner-Doudoroff pathway through 6-phosphogluconate
2-dehydro-3-deoxy-phosphogluconate aldolase activity
4-hydroxy-2-oxoglutarate aldolase activity
glutamine family amino acid biosynthetic process
Vitamin B6 metabolism
protein dimerization activity
NAD binding
4-phosphoerythronate dehydrogenase activity
pyridoxine biosynthetic process
Nicotinate and nicotinamide metabolism
glutaminase activity
NAD+ synthase (glutamine-hydrolyzing) activity
pyridine nucleotide biosynthetic process
NAD biosynthetic process
DNA topoisomerase activity
DNA topoisomerase type II (double strand cut, A...
DNA topoisomerase type II (double strand cut, A...
DNA metabolic process
DNA topological change
catalytic activity
porphobilinogen synthase activity
carbohydrate metabolic process
cation binding
metal ion binding
tetrapyrrole biosynthetic process
single-stranded DNA binding
DNA-(apurinic or apyrimidinic site) endonuclease activity
SOS response
DNA repair
DNA recombination
ATP-dependent activity, acting on DNA
RNA binding
ribosome
structural constituent of ribosome
cytosolic small ribosomal subunit
organellar small ribosomal subunit
cytosolic large ribosomal subunit
organellar large ribosomal subunit
translation
DNA-binding transcription factor activity
protein binding
Butanoate metabolism
Thiamine metabolism
Terpenoid backbone biosynthesis
RNA processing
methyltransferase activity
phosphoenolpyruvate-dependent sug...
FMN binding
maintenance of CRISPR repeat elements
membrane
rRNA binding
Cysteine and methionine metabolism
beta-Alanine metabolism
Lipopolysaccharide biosynthesis
Glycerolipid metabolism
regulation of transcription, DNA-templated
Oxidative phosphorylation
Methane metabolism
Propanoate metabolism
Reductive carboxylate cycle (CO2 fixation)
Phenylalanine metabolism
Isoquinoline alkaloid biosynthesis
transmembrane transport
transmembrane transporter activity
ATPase-coupled transmembrane transporter activity
ABC-type transporter activity
Oxidative phosphorylation 7.41%
Reductive carboxylate cycle (CO2 fixation) 7.41%
Isoquinoline alkaloid biosynthesis 7.41%
transmembrane transport 7.41%
ABC-type transporter activity 7.41%
